# Supplementary material for: Qualitative exploration of health professionals’ experiences of communicating positive newborn bloodspot screening results for nine conditions in England
Source: BMJ Open. 2020 Oct 1;10(10):e037081. doi: 10.1136/bmjopen-2020-037081 (PMC7534703; doi:10.1136/bmjopen-2020-037081)
Supplement: Author's manuscript [file bmjopen-2020-037081.draft_revisions.pdf]

BMJ Open

BMJ Open is committed to open peer review. As part of this commitment we make the peer review history of every article we publish publicly available.

When an article is published we post the peer reviewers' comments and the authors' responses online. We also post the versions of the paper that were used during peer review. These are the versions that the peer review comments apply to.

The versions of the paper that follow are the versions that were submitted during the peer review process. They are not the versions of record or the final published versions. They should not be cited or distributed as the published version of this manuscript.

BMJ Open is an open access journal and the full, final, typeset and author-corrected version of record of the manuscript is available on our site with no access controls, subscription charges or pay-per-view fees (http://bmjopen.bmj.com).

If you have any questions on BMJ Open's open peer review process please email info.bmjopen@bmj.com

BMJ Open

A qualitative exploration of health professionals' experiences of communicating positive newborn bloodspot screening results for nine conditions in England

Journal:	*BMJ Open*
Manuscript ID	bmjopen-2020-037081
Article Type:	Original research
Date Submitted by the Author:	17-Jan-2020
Complete List of Authors:	Chudleigh, Jane; City University, School of Health Sciences<br>Chinnery, Holly; St Mary's University Twickenham<br>Bonham, Jim; Sheffield Children's NHS Foundation Trust<br>Olander, Elinor ; City University London<br>Moody, Louise; Coventry University<br>Simpson, Alan; King's College London,<br>Morris, Stephen; University of Cambridge School of Clinical Medicine, Primary Care Unit<br>Ulph, Fiona; University of Manchester<br>Bryon, Mandy; Great Ormond Street Hospital For Children NHS Foundation Trust<br>Southern, Kevin; Institute of Child Health, Paediatrics
Keywords:	GENETICS, PAEDIATRICS, Cystic fibrosis < THORACIC MEDICINE, HAEMATOLOGY, Other metabolic, e.g. iron, porphyria < DIABETES & ENDOCRINOLOGY

SCHOLARONE™
Manuscripts

I, the Submitting Author has the right to grant and does grant on behalf of all authors of the Work (as defined in the below author licence), an exclusive licence and/or a non-exclusive licence for contributions from authors who are: i) UK Crown employees; ii) where BMJ has agreed a CC-BY licence shall apply, and/or iii) in accordance with the terms applicable for US Federal Government officers or employees acting as part of their official duties; on a worldwide, perpetual, irrevocable, royalty-free basis to BMJ Publishing Group Ltd ("BMJ") its licensees and where the relevant Journal is co-owned by BMJ to the co-owners of the Journal, to publish the Work in this journal and any other BMJ products and to exploit all rights, as set out in our licence.

The Submitting Author accepts and understands that any supply made under these terms is made by BMJ to the Submitting Author unless you are acting as an employee on behalf of your employer or a postgraduate student of an affiliated institution which is paying any applicable article publishing charge ("APC") for Open Access articles. Where the Submitting Author wishes to make the Work available on an Open Access basis (and intends to pay the relevant APC), the terms of reuse of such Open Access shall be governed by a Creative Commons licence – details of these licences and which Creative Commons licence will apply to this Work are set out in our licence referred to above.

Other than as permitted in any relevant BMJ Author's Self Archiving Policies, I confirm this Work has not been accepted for publication elsewhere, is not being considered for publication elsewhere and does not duplicate material already published. I confirm all authors consent to publication of this Work and authorise the granting of this licence.

For peer review only

TITLE PAGE

A qualitative exploration of health professionals' experiences of communicating positive newborn bloodspot screening results for nine conditions in England

Jane Chudleigh[1]*, Holly Chinnery[2], James R Bonham[3], Ellinor K Olander[1], Louise Moody[4], Alan Simpson[5], Steve Morris[6], Fiona Ulph[7], Mandy Bryon[8], Kevin W Southern [9]

*corresponding author

[1]Centre for Maternal and Child Health Research, City, University of London, EC1V 0HB, UK;

[2]Faculty of Sports, Health and Applied Science, St Mary's University Twickenham, TW1 4SX

[3]Division of Pharmacy, Diagnostics and Genetics Sheffield Children's NHS Foundation Trust, S10 2TH, UK;

4 Centre for Arts, Memory and Communities, Coventry University, CV1 5FB, UK;

[5] Florence Nightingale Faculty of Nursing, Midwifery & Palliative Care, King's College London SE1 8WA, UK; [6] University of Cambridge School of Clinical Medicine, CB2 0SP, UK; [7]Division of Psychology and Mental Health, The University of Manchester, M13 9PL, UK, [8]Paediatric Psychology and Play Services, Great Ormond Street Hospital for Children NHS Foundation Trust, WC1N 3JH, UK, [9] Women's and Children's Health, University of Liverpool, L69 3BX, UK

j.chudleigh@city.ac.uk; holly.chinnery@stmarys.ac.uk; jim.bonham@sch.nhs.uk; Ellinor.Olander@city.ac.uk; l.moody@coventry.ac.uk; alan.simpson@kcl.ac.uk; sm2428@medschl.cam.ac.uk; fiona.ulph@manchester.ac.uk; mandy.bryon@gosh.nhs.uk; kwsouth@liv.ac.uk

For peer review only - http://bmjopen.bmj.com/site/about/guidelines.xhtml

ABSTRACT

Objective: To explore health professionals (HPs) experiences of communicating positive NBS results, highlight differences, share good practice and make recommendations for future research.

Design: Qualitative exploratory design was employed using semi-structured interviews

Setting: Three National Health Service provider organisations in England

Participants: Seventeen health professionals involved in communicating positive NBS results to parents for all nine conditions currently included in the newborn bloodspot screening programme in England.

Results: Findings indicated variation in approaches to communicating positive newborn bloodspot screening results to parents, largely influenced by resources available and the lack of clear guidance. Health professionals emphasised the importance of communicating results to families in a way that is sensitive to their needs. However, many challenges hindered communication including logistical considerations, difficulty contacting the family and other health professionals, language barriers and parental reactions as well as resource considerations; training, and time.

Conclusion: Health professionals invest a lot of time and energy trying to ensure communication of positive newborn bloodspot screening results to families is done well. However, there continues to be great variation in the way these results are communicated to parents and this is largely influenced by resources available but also the lack of concrete guidance. How best to support health professionals undertaking this challenging and emotive task requires further exploration. We recommend evaluation of a more cohesive approach that meets the needs of parents and staff while being sensitive to the subtleties of each condition.

Trial registration: ISRCTN 15330120

Key words: Newborn bloodspot screening, communication

Word count: 5935

For peer review only - http://bmjopen.bmj.com/site/about/guidelines.xhtml

Strengths and limitations of this study

- Health professionals involved in the present study were employed in three different NHS organisations, increasing generalisability of the findings and were very experienced, supporting families for on average 10 years
- Not all health professionals in all three study sites were interviewed; the results may be biased towards those who had particularly strong views about communication of positive newborn bloodspot screening results.
- Health professionals were recruited from clinical teams involved in managing all nine conditions currently included in the NBS programme in England; previous work has mainly focussed on CF and SCD
- The study design, data collection and analysis were influenced by members of the patient and public involvement advisory group and relevant charities

For peer review only

For peer review only - http://bmjopen.bmj.com/site/about/guidelines.xhtml

INTRODUCTION

Newborn bloodspot screening (NBS) seeks to identify pre-symptomatic babies that are affected by genetic or congenital conditions[1]; early diagnosis leads to better health outcomes for the child[1 2]. In January 2015, NBS in England and Wales expanded and now covers nine conditions: sickle cell disease (SCD); cystic fibrosis (CF); phenylketonuria (PKU); homocystinuria (HCU); glutaric aciduria type 1 (GA1) medium chain acyl CoA dehydrogenase deficiency (MCADD), maple syrup urine disease (MSUD), isovaleric aciduria (IVA) and Congenital Hypothyroidism (CHT).

In the UK, parents are given information prior to and after birth and are asked to provide informed consent for their baby to take part in the NBS Programme. NBS involves a small amount of blood being taken from the baby's heel on day 5 of life. Each year, around 10,000 parents[1] of babies born in the UK are given a positive NBS result. This happens around 2-8 weeks after birth, depending on the condition[3 4]. Parents are informed of the NBS result, normally on the same day it becomes available, and are asked to bring their baby to a particular hospital for diagnostic testing within a given timeframe depending on the suspected condition.

National Guidance regarding the content and mode of communication between health professionals (HPs) and parents is generic and vague[1 5]. Communication of positive NBS results is often delivered by specialist nurses and is a subtle and skilful task which demands thought, preparation and evidence to minimise potentially harmful negative sequela[6-10]. However, training for this role is challenging due to the fact that for many of conditions, a positive results occurs relatively infrequently and may be communicated by a several members of the appropriate team people.

There is evidence of variations both nationally and internationally with regard to the approaches used to communicate positive NBS results particularly in relation to CF and SCD[10-13]. These variations include the different approaches used to communicate the results (such as in person during a home visit or by letter)[11 14], the choice of person or profession to deliver the result and the content of the communication[15-19].

[1] The term 'parents' refers to parents, carers or guardians.

For peer review only - http://bmjopen.bmj.com/site/about/guidelines.xhtml

The findings of Kai et al's study[14] informed the development of the current national guidelines for the communication process in the NBS programme[1 5], which recommend face-to-face communication by an appropriately trained HP. Despite these guidelines, a study reporting the findings from 67 interviews with parents about their experience of receiving CF or SCD carrier results following NBS indicated that disparity continues to exist regarding how the guidelines are implemented in practice[7]. The findings also indicated variability in the content and the way the result was communicated which led to increased parental anxiety and distress; the perceived lack of knowledge of the person communicating the result led to additional distress rather than the actual result *per se*[7]. This highlights the importance of understanding varied practice in order to identify, share and recommend good practice.

The purpose of this work was to explore HPs' experiences of communicating positive NBS results to highlight differences, share good practice and make recommendations for future research. The experiences of parents are reported elsewhere.

METHODS

A qualitative exploratory design was employed using semi-structured interviews as part of a large ongoing programme of work[20] . The study was approved by the London Stanmore ethics committee (17/LO/2102).

Setting

Study sites consisted of three National Health Service provider organisations in England that process comparable numbers of positive NBS reports annually for each of the nine conditions currently included in the NBS Programme. These consisted of two in Greater London and one in the West Midlands.

Patient and public involvement

Patient and public involvement (PPI) was instrumental in the design and conduct of this work. Parents of babies who had received a positive NBS screening result for one of the nine screened conditions formed a PPI advisory group who met prior to, during and following data collection. Their suggestions were incorporated into the study design, the data collection tools and the data analysis and presentation. In addition, views of representatives from charities for the screened conditions including Metabolic Support UK, the British Thyroid Foundation, the CF Trust and the Sickle Cell Society were also sought.

For peer review only - http://bmjopen.bmj.com/site/about/guidelines.xhtml

Inclusion and Exclusion Criteria

HPs involved in communicating positive NBS results in the previous 6 months were invited to take part in the study. HPs who had not been involved in communicating positive NBS results in the last 6 months or who had personal experience of receiving a positive NBS result were excluded.

Recruitment

Members of relevant clinical teams (medical consultants; general paediatricians; nurse specialists; specialist screening nurses) were initially identified through individual Trust websites and contacted via email and invited to participate. Identified HPs were asked if there were any other members of the clinical teams that the research team should contact to ensure views were representative. All potential participants were given the choice to participate or not and were reminded of their right to withdraw from the study at any time. Written informed consent was obtained from all participants ahead of the interview.

Data Collection

Semi-structured, face-to-face interviews comprising closed and open-ended questions were conducted by JC and HC from September 2018 to March 2019, to explore HPs experiences of communicating positive NBS to families. Topics covered included how HPs manage communication, examples of situations when it has gone well or not as well as expected and the possible reasons for this. We also explored how participants felt during the experience and asked for any suggested changes to practice. Interviews were audio recorded and transcribed verbatim.

Data Analysis

Interviews were analysed for themes[21]. An inductive approach to data analysis was used and themes generated using a latent approach[21] to provide a deeper understanding of approaches used to communicate positive NBS results to families. The six phases of thematic analysis described by Braun and Clarke[21] guided data analysis.

For peer review only - http://bmjopen.bmj.com/site/about/guidelines.xhtml

Two members of the research team (JC and HC) coded one interview transcript separately. These codes were then compared to inform and align code development[22] and a code book was developed. A further four transcripts were then coded separately by the same two members of the research team using the code book. These separately coded transcripts were then compared; inter-coder reliability was 84%. Following this, the same two members of the research team coded the remainder of the transcripts using the code book. Once this initial coding had been completed, all data for each code were compared to ensure consistency in coding and to enable the codes to be collapsed into themes. All quotes for each theme were collated to inform theme development. This was an ongoing, iterative process; new codes were developed and the definition of codes refined as analysis progressed.[23]

RESULTS

In total, 20 HPs were emailed and invited to participate. Two HPs did not respond to the invitation and one HP did not communicate the initial positive screening result and was therefore ineligible. Therefore, 16 face to face interviews were conducted with 17 HPs (two requested to be interviewed together); eight were from the West Midlands and the remaining nine were split across the two Greater London Trusts. Participants with experience of all the nine screened conditions were included. Interviews lasted on average 37 minutes (range 19-58 minutes). The sample consisted of 8 medical consultants, 1 medical registrar, 7 nurse specialists / advanced nurse practitioners and 1 screening nurse. Length of experience with newborn screening ranged from 2-38 years (mean 10 years)

Five themes were identified; communication between HPs, process of communicating with the family, parent and family centred care, availability of resources and challenges to effective communication. Illustrative quotations are used to support the themes.

Communication between HPs

The NBS card is used to collect the bloodspots from the baby and record relevant details about the baby and their family. HPs reported a range of communication approaches to ensure sufficient information was available to them prior to communicating with families. This started with the laboratory communicating the result to the relevant clinical team in a variety of ways. These included a letter, normally via email or a telephone call followed by

For peer review only - http://bmjopen.bmj.com/site/about/guidelines.xhtml

an email or a personal visit from a member of the screening laboratory to the clinical nurse specialist, the screening nurse, the on-call consultant or the named consultant depending on the condition, local resources and agreements.

"So we tend to find out from the newborn screening nurses. So, they'll be alerted by the labs and then they would give us [the physician] a call"[SS1P202]

The written initial communication consisted of a pro forma which was often developed locally and may or may not have been accompanied by a copy of the NBS card.

"...generally, we get a pro forma from the screening lab that's slightly different I think depending on the screening lab."[SS2P206]
"We actually started asking for the card."[SS2P201&02]

Receiving a copy of the NBS card was viewed favourably as it enabled HPs to check referral information and parental contact details if this were found to be ambiguous in any way.

Often, this would initiate a two pronged approach where HPs would commence gathering additional information about the child and family from health visitors (registered nurses or midwives who have undertaken additional training and work mainly with children from birth to 5 years and their families), midwives and/or primary care physicians before contacting the family.

"Sometimes, it is good to know, the family dynamics, social care issues etc. from somebody [the health visitor or midwife] who's already involved with the family". [SS1P203]

However, clinicians sometimes found it challenging to make contact with health visitors and/or midwives to gather additional information about the family.

"...quite often we are leaving messages to ask them [the health visitor or midwife] to call us back and quite often we are receiving those calls back, after we've already visited the family." [SS1P203]

For peer review only - http://bmjopen.bmj.com/site/about/guidelines.xhtml

For peer review only

Simultaneously, members of the multi professional team would also be contacted such as the physiotherapist, dietician or pharmacist (depending on the condition) to inform them that the child would be attending the hospital either the same or the following day.

"The CF nurse specialist would let other members of the team know, so the physio and dietician know that there was a new baby, positive screen so that they could be on standby, but not necessarily to see the family."[SS1P201]

Similarly, if the plan was to see the child in a local hospital rather than in a tertiary care centre, similar communication would happen between the specialist centre and local clinical teams.

Process of communicating with the family

Once sufficient information had been gathered about the child and family and disseminated appropriately to ensure members of the team were available to see the child and family if needed, attention turned to communicating with the family. The initial contact with the family was undertaken in a variety of ways by different members of the clinical team. This included, face to face contact in the family's home, via telephone, text message or letter from the screening nurse, relevant clinical nurse specialist or medical consultant. Some respondents commented that they felt the person who told the family should not be a member of the specialist clinical team who would go on to care for the child and they felt this may taint any ongoing relationship. Others felt it was important that the person who gave the initial positive NBS result should be part of the child's clinical team to start building familiarity and continuity of care or that there should be someone present who is known to the family.

So the ideal is that either the midwife or the health visitor can come on the visit so that somebody in the family already knows. [SS1P203]

Views regarding how the initial communication with the family was conducted varied. Some clinicians felt it should be face-to face as it allowed the clinician to gather information about the family that would help to inform the follow-up visit at the hospital with the clinical team.

For peer review only - http://bmjopen.bmj.com/site/about/guidelines.xhtml

You just don't know what's going on in the home environment and you've, sort of, been and witnessed it for yourself and it just gives you a good insight into the family dynamics or what's going on or what support mechanism are in place. You can't get that over the telephone." [SS1P202].

Others felt that a telephone call would be more appropriate as they felt families should be told as soon as possible while some felt that a text message asking the family to call them back had worked really well and that a home visit may be quite intrusive. This suggests that sometimes, the approach to communication may not be steered wholly by the needs of the family but sometimes the experience of the person communicating the result.

"You can't really get rid of anyone in someone's house, can you? …You'd be a bit like, 'Right, go now from my house. Get out of my house'...how scary must it be for somebody to turn up on your doorstep"[SS2P201&02]

All respondents stated that when introducing themselves to the family during the initial communication of the positive NBS results, they would not disclose their clinical speciality but would refer to themselves as a specialist nurse or doctor from the hospital. Clinicians stated this was because they did not want to name the condition before they had explained to the family that they were discussing a screening result rather than a diagnosis. Regardless of who or how the initial communication took place, all respondents acknowledged the importance of 'getting it right'. Respondents felt it was important the person giving the positive screening result to the family was knowledgeable about the condition they would be discussing with the family. Indeed, many felt this was more important than how the information was delivered and could influence perceptions of ongoing care.

"I think the most important thing, in my opinion, is that the person giving that information, the first time, needs to be someone that can answer some questions." [SS1P207]

"I think that first telephone call and the first time you see them is absolutely critical…I think the family's views of what's going to come next will be completely modified by how it's done and their confidence in the service." [SS2P206]

For peer review only - http://bmjopen.bmj.com/site/about/guidelines.xhtml

However, it was also acknowledged that although various guidelines and protocols existed for the laboratory staff when processing the NBS card and for treatment and management once the child had been diagnosed, there was a paucity of guidance regarding communication of the initial positive NBS result.

"We've got all the protocols around timelines and KPIs etc. but the one bit we don't have anything concrete about is who breaks the news, what level of training or experience they need to have before they do it and what the expectation is of what they should cover in that visit." [SS1P203]

Consequently, the content of the initial communication also varied considerably. Some clinicians spoke about having a template they followed which helped them to ensure they imparted all the information required during what could sometimes be a very emotive interaction. Most agreed that they would try to keep the information about the suspected condition during the initial communication quite brief due to the fact it is a screening result and therefore it would not be appropriate to give too much information about a condition that had not been confirmed. In addition, families were often perceived as being unable to absorb the information very well due to the shock of an unexpected result. Finally, clinicians knew that when families are seen the same or following day, they would receive a lot more information and therefore were reluctant to overload them with information during the first contact.

"So we always have said, 'Screening will be rechecked. The bloods will be redone.'" [SS1P203]
"We don't usually give them much information, they're [the family] usually really upset." [SS2P201&02]

However, HPs did recognise the importance of signposting families to additional information sources such as charity websites and the National NBS website following the initial communication and gave examples of excellent practice.

"We always take out suspected leaflets. They always get a copy of something to read. We take out a map for the hospital…so they know where to go to in that building….We leave them a letter that confirms what the screening result is and what that might mean for the baby. On

For peer review only - http://bmjopen.bmj.com/site/about/guidelines.xhtml

the top of that letter, it has our mobile number and office number to give them permission to ring us if they were at all worrying."[SS1P203]

Parent and family centred care

The importance of having a parent and family-centred approach when communicating the positive NBS result was emphasised by all clinicians and was considered to be an example of excellent practice. HPs spoke about the importance of the content of the initial communication. This was subdivided into a beginning, namely, remembering to congratulate parents on the birth of their child. This was followed by a section focussed on tailoring the quantity and level of information given depending on the parental response, not giving too much information or overwhelming parents, being honest and providing emotional support and reassurance where needed.

"So, you just have to judge how much information to give them and how best to support them because if parents are crying and upset about the diagnosis, you obviously have to support them in a different way to other parents." [SS1P205]

Finally, ending with a positive message, giving parents' time and making sure the family know that the baby's mum should not attend the first appointment at the hospital on her own. Also, the importance of treating each family as individuals and acknowledging the enormity of the task they are undertaking.

"I think it's easy, particularly if you're tired or something, to appear a bit more routine, run of the mill, whereas, obviously, it's a really big thing. This is their child, and this is an important piece of information, and it matters hugely to them. You have to try and reflect that in how you talk to them, rather than them just being another parent of another sickle baby, which is the danger."[SS3P206]

Some clinicians said they endeavour to find out how much parents already know for instance if they have another child with the condition or if they have been searching the internet before embarking on any explanations. Also, gauging how much families want to know as well as how much families are actually absorbing and when it is time to stop.

For peer review only - http://bmjopen.bmj.com/site/about/guidelines.xhtml

"…there are some families that come in that want to know every single detail about everything and you try and be systematic in how you deliver that information. There are other parents that you can tell, although you're trying to tell them things, they are not really taking it on board." [SS1P205]

Finally, HPs recognised the importance of supporting the whole family and therefore not making it the responsibility of the parents to share information or educate other family members.

"We're there to support whoever. The home visit follow-up that I do, I say, 'All are welcome, you know, if granny and granddad want to be there, if they've got questions, I'm happy to speak with them.' Very often, grandparents, aunts and uncles are there, you know. I've been to one and people have been there on mass, but that's their family support network and if that's usually there in place, we don't exclude anyone. I'm happy to talk to any family member." [SS1P202]

Availability of resources

HPs at all levels and for all conditions acknowledged a lack of training and related competencies in terms of breaking bad news to families. This resulted in screening nurses in particular developing their own training programme to address this deficit.

"…we've had to develop some competencies….but because it's so unique and there aren't other nurses funded through a lab post, that there aren't competencies around." [SS1P203]

Resources available at different hospitals also influenced communication of the initial positive NBS result. Therefore, even if clinicians felt that offering to do a home visit to deliver the initial positive NBS result would be beneficial, the need to prioritise resources often meant this would not be an option.

"…we don't have the capacity [to do home visits]….I think it will be good if we can do that, but we definitely don't have the capacity for that." [SS2P203]

One hospital had screening nurses and part of their role was to deliver the initial positive NBS result to families, usually face-to-face in their home. In the other two hospitals, this

For peer review only - http://bmjopen.bmj.com/site/about/guidelines.xhtml

resource was not available and therefore the responsibility of delivering the initial positive NBS result to families stayed with the relevant clinical team. Even though HPs who were able to, felt very positive about the ability to offer home visits, these were resource intensive particularly when compared to the option to contact parents via telephone.

"Probably around 40 minutes is a quick visit. The longest visit I have been there [excluding travel] is probably about three hours."[SS1P203]

The ability to offer timely follow-up appointments with the clinical team both due to clinical need and also to alleviate parental anxiety was also viewed as important. However, many respondents discussed the availability of resources as a potential barrier to this and this seemed particularly evident for babies with SCD.

"It can be a week if we've got a slot, or it can be anything up to four to five weeks….I think it is a very stressful time and some families find that too long." [SS1P209]

For other conditions, clinical need meant that families were seen almost immediately by the clinical team after they had been given the initial positive NBS result.

"So, it's all clinically indicated….the rest of the metabolic conditions will be seen the same day." [SS1P203]

Although ensuring families are seen quickly after being told their child has a positive NBS result was seen as important by the clinical team, this also posed problems on occasions in terms of the financial burden for families needing to travel to the hospital at short notice. Currently, there is no budget to prospectively pay for families' travel expenses to attend clinic appointments which means that families are expected to meet these costs in the first instance. Although it may be possible, in certain circumstances, to apply to have these reimbursed at a later date, this does not help families who struggle to pay these costs up front.

"…but we have had families who, it has happened where a family said, 'I just don't think I have the money. I can't afford to come' and saying, 'We'll pay you back' doesn't help and that is difficult [SS2P206].

For peer review only - http://bmjopen.bmj.com/site/about/guidelines.xhtml

Some clinicians also felt that the setting within which the family met the clinical team was important in terms of first impressions.

"This room was designed that it hasn't got a computer or a phone, unlike our other consultation rooms. So, it's a quiet space to deliver the news to the family." [SS1P202]

Availability of resources for diagnostic testing was also viewed as potentially problematic.

"…when we started, the labs were kitted out, such that they would be able to offer a sweat test and a result the same day, any day….because of cuts…they're much less flexible in terms of what they can offer." [SS1P201]

Therefore, although HPs felt very strongly about offering a parent-centred approach to communicating positive NBS results to parents, availability of training, staff and physical space could act as a barrier.

Other concerns related to practical and resource considerations included adding new conditions to the NBS Programme and changes in geographical areas covered by screening laboratories particularly in relation to inherited metabolic diseases and CHT.

"…they [babies with CHT] are dealt with by fifteen different centres…and I think that causes the labs quite a lot of problems. The labs would much prefer fewer centre." [SS2P207]

Challenges to effective communication

HPs in the current study were asked about their experience of communicating positive NBS result to families. Several staff commented on the personal and emotional impact of this aspect of their role.

"Some can be pretty traumatic and you just feel like you've destroyed their world." [SS1P202]

"It's very emotional for me sometimes, very, very emotional…the mother, a new born, holding a new born baby crying…based on the result you gave them…you wish you could change the result for them." [SS3P211]

For peer review only - http://bmjopen.bmj.com/site/about/guidelines.xhtml

Despite this, no formal mechanisms were in place to support HPs. However, all reported that they had developed their own support mechanisms within their teams.

"…everyone's very used to doing it, so everyone completely gets it and completely understands….you have a bit of a debrief with the consultant who's seeing the family." [SS3P202]

HPs also struggled to make contact with parents for a variety of reasons including, the contact details on the NBS card being illegible, parents having moved or staying with relatives after the NBS sample was taken and parents not answering their phones.

" You fill in clearly….not flipping scribbled…the name of the person, a first number, a second number of a partner or someone like that…even though we had a picture of the card, they hadn't filled in any of the details" [SS2P201&02]

"It's not being able to get in contact with people, because they're not picking up on a random London number…The amount of junk calls we all get nowadays, these people are just not picking up the phone." [SS2P201&02]

Once parents had been contacted, other challenges arose related to parental attributes. A common theme related to communicating with parents who did not speak English as a first language. This was not always apparent until after the clinician had attempted to make contact with the parent. This led to parents not understanding; what was being communicated, the seriousness of what was being communicated or simply not engaging in the conversation due to lack of understanding.

"If English isn't the first language, I certainly find it much more difficult to reassure, and try to be empathetic because you're worried about just getting the basic understanding across." [SS2P206]

Managing families who do not believe the diagnosis either because their child has no signs or symptoms or due to religious or cultural beliefs was also difficult. Such beliefs may also affect engagement with recommended medication and/or treatments. This was particularly evident for those families with a child with SCD.

For peer review only - http://bmjopen.bmj.com/site/about/guidelines.xhtml

"They just wouldn't accept that they'd got sickle, because they looked well." [SS1210]

"…so you get a few that either don't believe the diagnosis or their cultural beliefs are that God will mean that they don't have to do anything medical that we suggest." [SS1P205]

Clinicians commented that this often led to patients not attending clinical appointments which also posed challenges in terms of monitoring.

Oher challenges related directly to the NBS Programme. The NBS Programme is designed to identify babies with a higher chance of being affected by one of the screened conditions, often before they are symptomatic, so they can start treatment as soon as possible if needed, and therefore improve outcomes. However, the need to inform parents that their baby has a positive NBS result for a potentially life changing and/or life limiting condition when the baby is asymptomatic was challenging.

"You come to see the doctor because your child is unwell, whereas, this is the opposite, in that, you've got a child who appears perfectly healthy, and you're telling them that they're unwell." [SS1P207]

DISCUSSION

This study furthers our understanding of HPs' experiences of communicating positive NBS results to families. It is clear that staff involved in communication of positive NBS results are passionate about making sure that although the message is distressing for parents, it is done well. Variation in communication practice continue to exist and is influenced by many factors including resources available but also the lack of clear guidance. This impacted on the methods used to communicate positive NBS results but also the content of the communication to parents. This is supported by previous research which has been conducted both nationally and internationally[7 10 11 13] and suggests that further guidance may be needed to ensure a more cohesive approach which meets the needs of parents' and staff while being sensitive to the subtleties of each condition. However, the issue of finite resources and the need to prioritise these also needs careful consideration.

For peer review only - http://bmjopen.bmj.com/site/about/guidelines.xhtml

An overarching message from the HPs involved in the present study was the desire to ensure communicating positive NBS result to families is parent and family-centred. Once HPs were aware of a positive NBS result, they spent a great deal of time 'setting the scene' by gathering information from various sources in preparation for speaking with the family and organising the follow-up appointment with the clinical team. Often this important and necessary work would be time consuming and labour intensive in terms of identifying individuals who either obtained the original sample (midwives) or might have additional information about family dynamics (health visitor) and may be hindered by poor completion or lack of information on the NBS card.

Current guidance[1 5] does not explicitly state who is the 'right' person to communicate a positive NBS result to families or what training or qualifications they should have and specific training to undertake this role is not available. While this does allow flexibility in terms of resources, it can lead to disparity in terms of parental experience of receiving the NBS result. There is no specific guidance regarding exactly what information should be shared with parents during the initial communication[5]. While some HPs alluded to using an informal checklist, this was not universal and therefore may not lead to consistency between clinicians. However, clinicians did recognise the importance of the person imparting the result, having adequate condition specific knowledge, this is consistent with previous research[15 16 19]. These are common problems that have been highlighted both nationally and internationally.[13] As these are not dependent on specific healthcare systems *per se*, the findings of the present study could also be extrapolated to screening programmes in other countries.

Clinicians experienced many challenges which hindered the communication of positive NBS results to families. This often stemmed from inadequate information on the NBS card but also parental reactions which could hinder effective communication. When parents are told the NBS result, their baby is often pre-symptomatic as this is one of the purposes of the NBS Programme which means the result is often unexpected.[10 17 18] This can make it difficult for parents to accept their baby may have an underlying health condition which can impact on treatment adherence and affect attendance at follow-up appointments. In addition, parental religious or cultural beliefs could also impact on parent's acceptance of their baby's suspected condition.[10 24] These results demonstrate the importance of always recording clear contact information for all relevant family members on the NBS card as well information

For peer review only - http://bmjopen.bmj.com/site/about/guidelines.xhtml

about the language spoken, the need for a translator and any relevant religious or cultural information.

The impact on HPs of communicating of positive NBS results to families has rarely been considered. It has been acknowledged that the emotional management of families could lead to additional stress and anxiety.[25] However, HPs in the present study stated they found communicating positive NBS results to families difficult and emotive yet there were no formal mechanisms in place to support them. Despite this, HPs said they felt well supported by their colleagues. However, given the high levels of stress being reported by nurses and doctors and the reported rates of suicide amongst these professions[26 27] perhaps more consideration needs to be given to support staff undertaking such emotionally charged endeavours.

Strengths and Limitations

The current study has numerous strengths. HPs involved in the present study were employed in three different trusts; two in London and one in Birmingham and were very experienced, supporting families for on average 10 years. Clinicians with less experience may have felt even more strongly about the need for clearer guidance. In addition, HPs were recruited from clinical teams involved in managing all of the conditions currently included in the NBS programme. This increases the generalisability of the study findings as previous work has mainly focussed on CF and SCD. In addition, the study design, data collection and analysis were influenced by members of the PPI advisory group and relevant charities.

While the HPs interviewed in the present study represented those who care for children and families with all of the conditions currently included in the NBS Programme in England and Wales, not all HPs in all three sites were interviewed (17/20, 85%). Therefore, the results may be biased towards those who had particularly strong views about communication of positive NBS results.

The researchers are experienced in this field which may have biased data collection and analysis. Following the interviews, a HP event was held and the initial findings of the interviews were presented to the HPs who had been interviewed in each site. The purpose of

For peer review only - http://bmjopen.bmj.com/site/about/guidelines.xhtml

this was to ensure that the analysis accurately represented their views. Participants agreed that the analysis was correct and priorities identified were accurate.

Recommendations for practice

The findings from this study suggest a number of recommendations for practice. For example, development of a competency framework for individuals involved in the process of communicating positive NBS results to families would ensure only HPs who are appropriately prepared undertake this task.

In addition, development of a standard laboratory form for communicating positive NBS results to clinical teams would ensure that when results are received from several laboratories by one clinical team, the information provided by each is consistent.

The development of condition specific checklists for HPs involved in communicating positive NBS result to families would ensure that vital information is consistently relayed to families and less experienced staff would be supported in terms of the information they need to provide. These could also act as an aide memoir for HPs as it is know that this can be a very distressing time for parents and so it would help them to remain focussed. In addition, this would ensure that clear contact information for all relevant family members including information about language spoken, translation needs and religious or cultural requirements could also be recorded and would be easily accessible for all member of the child and family's care team.

Guidance regarding reliable sources of further information for parents would also reduce alarm that can be caused by accessing unhelpful content on the internet immediately after the initial communication of the positive NBS result. This might include the use of specifically designed Apps or other forms of "easy to access" and helpful on-line information for parents.

Finally, regular clinical supervision and emotional support for all staff engaged in such work, should be encouraged to ensure staff are adequately supported to undertake this challenging task.

For peer review only

CONCLUSION

HPs invest a lot of time and energy trying to make sure communication of positive NBS results to families is done well. However, there continues to be great variation in the way positive NBS results are communicated to parents and this is largely influenced by resources available but also the lack of concrete guidance. Evidence based guidance derived from HPs' and parents' perspectives is needed to ensure there is parity in terms of communication practices. These should include, what training should be undertaken prior to undertaking this challenging role and guidelines related to what information should be relayed by whom and when. In addition, adequate support mechanism for HPs should be in place.

For peer review only

Acknowledgements: We would like to thank the Newborn Screening Laboratory Directors in England for agreeing to act as local principle investigators for this study as well as members of clinical teams who gave their valuable time. We would also like to thank all the parents in the Public and Patient Involvement Advisory Group for this study for their invaluable input.

Author Contributions: All authors were involved in the design and conceptualisation of the study. JC and HC collected and analysed data. All authors contributed to and approved the final manuscript.

Funding: This study was supported by the National Institute for Health Research (NIHR) [Health Services and Delivery Research (project reference 16/52/25)]. The views expressed are those of the authors and not necessarily those of the NIHR or the Department of Health and Social Care.

Competing interests: None declared.

Ethics approval: This study was approved by the London Stanmore ethics committee, reference 17/LO/2102

Data sharing statement: Data are available upon reasonable request from the corresponding author subject to restrictions to preserve anonymity and personal privacy [JC]. These data are not publicly available as they contain information that could compromise research participant privacy/consent. Data will be available beginning 1 year and ending 5 years after publication to researchers who propose a methodologically sound proposal. Proposals should be directed to j.chudleigh@city.ac.uk. To gain access, data requesters will need to sign a data access agreement.

For peer review only - http://bmjopen.bmj.com/site/about/guidelines.xhtml

REFERENCES

1. UK Newborn Screening Programme Centre. Health Professional Handbook: A guide to newborn blood spot screening for healthcare professionals. London: UK Newborn Screening Programme Centre, 2012:1-56.
2. Bush A. Newborn screening for cystic fibrosis – benefit or bane? *Paediatric respiratory reviews* 2008;9:301-2.
3. Public Health England. Newborn Blood Spot Screening Programme in the UK: Data Collection and Performance Analysis Report 2016 to 2017. London, 2018:1-65.
4. Public Health England. NHS Sickle Cell and Thalassaemia Screening Programme Data Report 2015/16: Trends and performance analysis. London, 2017:1-81.
5. Public Health England. Newborn blood spot screening: programme handbook: Public Health England, 2018.
6. Salm A, Yetter E, Tluczek A. Informing parents about positive newborn screening results: Parents' recommendations *Journal of Child Health Care* 2012;16(4):367-81.
7. Ulph F, Cullinan T, Qureshi N, et al. Parents' responses to receiving sickle cell or cystic fibrosis carrier results for their child following newborn screening. *Eur J Hum Genet* 2015;23(4):459-65. doi: DOI 10.1038/ejhg.2014.126
8. Ulph F, Cullinan T, Qureshi N, et al. The impact on parents of receiving a carrier result for sickle cell or cystic fibrosis for their child via newborn screening. *Eur J Hum Genet* 2014;22
9. Tluczek A, Clark R, McKechnie AC, et al. Factors affecting parent-child relationships one year after positive newborn screening for cystic fibrosis or congenital hypothyroidism. *J Dev Behav Pediatr* 2015;36(1):24-34. doi: 10.1097/DBP.0000000000000112
10. Chudleigh J, Buckingham S, Dignan J, et al. Parents' Experiences of Receiving the Initial Positive Newborn Screening (NBS) Result for Cystic Fibrosis and Sickle Cell Disease. *J Genet Couns* 2016 doi: 10.1007/s10897-016-9959-4
11. Parker H, Qureshi N, Ulph F, et al. Imparting carrier status results detected by universal newborn screening for sickle cell and cystic fibrosis in England: a qualitative study of current practice and policy challenges. *BMC Health Serv Res* 2007;7:203. doi: 1472-6963-7-203 [pii] 10.1186/1472-6963-7-203 [published Online First: 2007/12/15]
12. Ulph F, Cullinan T, Qureshi N, et al. Parents' responses to receiving sickle cell or cystic fibrosis carrier results for their child following newborn screening. *Eur J Hum Genet* 2015;23(4):459-65. doi: 10.1038/ejhg.2014.126
13. Chudleigh J, Ren CL, Barben J, et al. International approaches for delivery of positive newborn bloodspot screening results for CF. *Journal of cystic fibrosis : official journal of the European Cystic Fibrosis Society* 2019;18(5):614-21. doi: 10.1016/j.jcf.2019.04.004
14. Kai J, Ulph F, Cullinan T, et al. Communication of carrier status information following universal newborn screening for sickle cell disorders and cystic fibrosis: qualitative study of experience and practice. *Health Technol Assess* 2009;13(57):1-82, iii. doi: 10.3310/hta13570 [published Online First: 2009/12/02]
15. Tluczek A, Koscik RL, Farrell PM, et al. Psychosocial risk associated with newborn screening for cystic fibrosis: parents' experience while awaiting the sweat-test appointment. *Pediatrics* 2005;115(6):1692-703. doi: 10.1542/peds.2004-0275
16. Collins JL, La Pean A, O'Tool F, et al. Factors that influence parents' experiences with results disclosure after newborn screening identifies genetic carrier status for cystic fibrosis or sickle cell hemoglobinopathy. *Patient Educ Couns* 2012 doi: S0738-3991(11)00606-9 [pii] 10.1016/j.pec.2011.12.007 [published Online First: 2012/01/14]
17. Buchbinder M, Timmermans S. Newborn screening for metabolic disorders: parental perceptions of the initial communication of results. *Clin Pediatr (Phila)* 2012;51(8):739-44. doi: 0009922812446011 [pii] 10.1177/0009922812446011 [published Online First: 2012/05/09]

For peer review only - http://bmjopen.bmj.com/site/about/guidelines.xhtml

18. DeLuca JM, Kearney MH, Norton SA, et al. Parents' experiences of expanded newborn screening evaluations. *Pediatrics* 2011;128(1):53-61. doi: peds.2010-3413 [pii] 10.1542/peds.2010-3413 [published Online First: 2011/06/29]
19. Rueegg CS, Barben J, Hafen GM, et al. Newborn screening for cystic fibrosis - The parent perspective. *Journal of cystic fibrosis : official journal of the European Cystic Fibrosis Society* 2016;15(4):443-51. doi: 10.1016/j.jcf.2015.12.003
20. Chudleigh J, Bonham J, Bryon M, et al. Rethinking Strategies for Positive Newborn Screening Result (NBS+) Delivery (ReSPoND): a process evaluation of co-designing interventions to minimise impact on parental emotional well-being and stress. *Pilot Feasibility Stud* 2019;5:108. doi: 10.1186/s40814-019-0487-5
21. Braun V, Clarke V. Using thematic analysis in psychology. *Qualitative Research in Psychology* 2006;3(2):77-101.
22. Milford C, Kriel Y, Njau I, et al. Teamwork in Qualitative Research: Descriptions of a Multicountry Team Approach. *International Journal of Qualitative Methods* 2017;16:1-10.
23. DeCuir-Gunby JT, Marshall PL, McCulloch AW. Developing and Using a Codebook for the Analysis of Interview Data: An Example from a Professional Development Research Project. *Field Methods* 2011;23(2):136-55.
24. Marsh VM, Kamuya DM, Molyneux SS. 'All her children are born that way': gendered experiences of stigma in families affected by sickle cell disorder in rural Kenya. *Ethn Health* 2011;16(4-5):343-59. doi: 10.1080/13557858.2010.541903
25. Moody L, Atkinson L, Kehal I, et al. Healthcare professionals' and parents' experiences of the confirmatory testing period: a qualitative study of the UK expanded newborn screening pilot. *BMC Pediatr* 2017;17(1):121. doi: 10.1186/s12887-017-0873-1
26. Gerada C. Doctors and suicide. *Br J Gen Pract* 2018;68(669):168-69. doi: 10.3399/bjgp18X695345
27. Selby A. More than 300 overworked NHS nurses have died by suicide in just seven years. *Daily Mirror* 2019.

For peer review only

For peer review only - http://bmjopen.bmj.com/site/about/guidelines.xhtml

Chudleigh *et al. Pilot and Feasibility Studies* (2019) 5:108
https://doi.org/10.1186/s40814-019-0487-5

Pilot and Feasibility Studies

STUDY PROTOCOL **Open Access**

Rethinking Strategies for Positive Newborn Screening Result (NBS+) Delivery (ReSPoND): a process evaluation of co-designing interventions to minimise impact on parental emotional well-being and stress

Jane Chudleigh[1*], Jim Bonham[2], Mandy Bryon[3], Jill Francis[4], Louise Moody[5], Steve Morris[6], Alan Simpson[7], Fiona Ulph[8] and Kevin Southern[9]

Abstract

Background: Newborn blood spot (NBS) screening seeks to prevent ill health, disability and death through early diagnosis and effective intervention. Each year, around 10,000 parents of babies born in England are given a positive NBS result indicating their child may be affected or carriers of one of the nine conditions currently screened for. Despite guidance, these results are inconsistently delivered to parents across geographical regions. There is evidence that many parents are dissatisfied with how NBS results are communicated to them and that poor communication practices can lead to various negative sequelae. The purpose of this study is to co-design, implement and undertake a process evaluation of new, co-designed interventions to improve delivery of initial positive NBS results to parents.

Methods: This mixed-methods study will use four phases with defined outputs. Family Systems Theory will form the theoretical basis for the study. The principles and methods of experience-based co-design will underpin intervention development. Normalisation Process Theory will underpin the process evaluation of the interventions co-designed to improve the delivery of positive NBS results to parents. An economic analysis will determine resource use and costs of current practice and of implementing the new co-designed interventions. The nominal group technique will be used to inform the selection of suitable outcome measures for a future evaluation study.

Discussion: The main output of the proposed study will be co-designed interventions for initial communication of positive NBS results to parents ready to be evaluated in a definitive evaluation study.

The interventions, co-designed with parents, will help to minimise potential negative sequelae associated with poor communication practices by considering parental and staff experiences as well as healthcare challenges such as finite resources. In addition, information about indicative costs associated with different communication strategies will be determined.

It is anticipated it may also be possible to extrapolate principles of good communication practices from the present

(Continued on next page)

* Correspondence: j.chudleigh@city.ac.uk
[1]Centre for Maternal and Child Health Research, City, University of London, London EC1V 0HB, UK
Full list of author information is available at the end of the article

© The Author(s). 2019 **Open Access** This article is distributed under the terms of the Creative Commons Attribution 4.0 International License (http://creativecommons.org/licenses/by/4.0/), which permits unrestricted use, distribution, and reproduction in any medium, provided you give appropriate credit to the original author(s) and the source, provide a link to the Creative Commons license, and indicate if changes were made. The Creative Commons Public Domain Dedication waiver (http://creativecommons.org/publicdomain/zero/1.0/) applies to the data made available in this article, unless otherwise stated.

(Continued from previous page)
study for the delivery of bad news to parents for children newly diagnosed with other conditions including cancer and other chronic conditions such as diabetes or epilepsy.

Trial registration: ISRCTN 15330120 date of registration 17/01/2018

Keywords: Newborn bloodspot screening, Cystic fibrosis, Sickle cell disease, Metabolic, Congenital hypothyroid

Background

Newborn bloodspot screening (NBS) in the UK involves obtaining parents' informed consent to take a small sample of blood from their baby's heel (heel prick) on day 5 of life to determine if the baby may be affected by one of nine life-changing conditions[1]. These conditions are as follows: sickle cell disease (SCD), cystic fibrosis (CF), congenital hypothyroidism, phenylketonuria, medium-chain acyl-CoA dehydrogenase deficiency, maple syrup urine disease, isovaleric acidaemia, glutaric aciduria type 1 and homocystinuria (pyridoxine unresponsive). A positive NBS (NBS+) result indicates that the baby may be affected or be a carrier of one of these conditions and will often require further diagnostic testing before a definitive diagnosis is made [1].

Each year in England, over 10,000 parents are informed of their child's NBS+ result around 2–8 weeks, depending on the condition, after birth [2, 3]. Most babies with initial NBS+ results for SCD and approximately half of those with an NBS+ result for CF will later be confirmed as gene carriers but unaffected by the disease. However, over 1300 babies will eventually be diagnosed as being affected by one of the conditions currently screened for [2, 3].

Variation in communication practice in the UK

There is evidence of regional variations in the UK with regard to the approaches used to communicate NBS+ results and, in particular, suspected carrier status for CF and SCD following NBS. These approaches include receiving the result by letter to in-person communication during a home visit [4, 5]. The findings of Kai et al.'s study [5] informed the development of the current national guidelines for the communication process in the NBS Programme (NBSP), which recommend face-to-face communication by an appropriately trained health professional [1]. Despite these guidelines, a recent study with parents exploring their experience of receiving CF or SCD carrier results following NBS indicated that disparity continues to exist regarding how the guidelines are implemented in practice [6].

[1]https://www.gov.uk/government/collections/newborn-blood-spot-screening-programme-supporting-publications

Impact of poor communication practices

Poor, or inappropriate, communication strategies for NBS+ can influence parental outcomes in the short term [6–11] and may also have long-term impact [12]. Evidence suggests the distress caused can manifest in several ways including arguments between couples including apportioning of blame [6, 9, 13], alteration of life plans and inability to conduct tasks of daily living such as going to work or socialising [6], long-term alterations in parent-child relationships [12] and mistrust and lack of confidence affecting ongoing relationships with staff [9]. There is also evidence of increased parental distress resulting in parents reducing their child's interaction with others, particularly in the case of CF [6]. Parents also experienced poor intra- and interpersonal relationships within their family system and more widely [14].

With the expansion of the NBSP in the UK in 2015 and further future growth planned [15], it is timely to ensure that clinical advantages of this process continue to outweigh potential long term negative psychosocial consequences for the families involved. It is essential that approaches used to deliver this information to parents are informed by them and shaped to meet their needs. It may not be possible to remove parental distress completely from what is an upsetting time. However, it is important for staff to communicate NBS+ results in a manner that minimises distress to families and does not detrimentally affect parents' relationships with their child and other family members. Empirical evidence is lacking on the potential impact of information provision on parental well-being and decision-making strategies. As finite budgets are available to provide communication strategies on a national level, there is a need to understand both the short and long-term costs of different aspects of the NBSP including the implications of providing NBS+ results. A further consideration is ensuring parents are informed well enough to facilitate communication within and between family members.

The aim of this study is to work with parents to co-design, implement and undertake a process evaluation (including cost analysis) of new interventions to improve the delivery of initial NBS+ results to parents.

Methods

Design

The theory underpinning the proposed study is Family Systems Theory [16] because of the potential vulnerability

For peer review only - http://bmjopen.bmj.com/site/about/guidelines.xhtml

of family relationships if the initial NBS+ information is not shared as effectively and empathetically as possible [17]. This mixed-methods study will use four phases with defined outputs and will be guided by the Medical Research Council Complex Interventions Framework [18]. A description of the phases and the study design can be seen in Fig. 1. The principles and methods of experience-based co-design (EBCD) will underpin intervention development [19–25]. Normalisation Process Theory [26, 27] will underpin the process evaluation of the new, co-designed interventions to improve the delivery of NBS+ results to parents. An economic analysis will be undertaken to determine resource use and costs of current practice and implementing the new co-designed interventions. The process evaluation will be used to explain discrepancies between expected and observed outcomes, contribute to understanding how context influences those outcomes and provide insights to aid planning of the future evaluation study. The economic analysis will provide additional data that will also be useful to stakeholders and decision makers [18]. The nominal group technique [28, 29] will be used to plan the future evaluation study including selection of suitable outcome measures.

The nine conditions currently screened for by the NBSP will be grouped into four condition-specific groups (CSGs) based on the urgency with which communication of the NBS+ result should occur (Table 1).

Setting

In England, there are 13 newborn screening laboratories that process the results for the nine conditions that are currently included in the NBSP, and these will comprise the study sites.

Inclusion and exclusion criteria

For all study phases, parents of children who have received a NBS+ result in the previous 3–12 months including true positives, false positives and children who are diagnosed as 'cystic fibrosis screen positive, inconclusive diagnosis' will be included in the study. Parents of children who have received a negative NBS result or with children with co-morbidities that are likely to influence their perception of receiving their NBS+ result and parents whose recruitment is contraindicated on psychosocial grounds (identified by their health visitor or specialist nurse) will be excluded. Parents who are unable to understand and give informed consent will also be excluded.

For all study phases, staff employed in NBS laboratories and involved in the processing of NBS+ results and staff who have been involved in communicating NBS+ results to parents in the last 6 months will be included. Staff who have not been involved in processing NBS+ results or communicating NBS+ results to parents in the

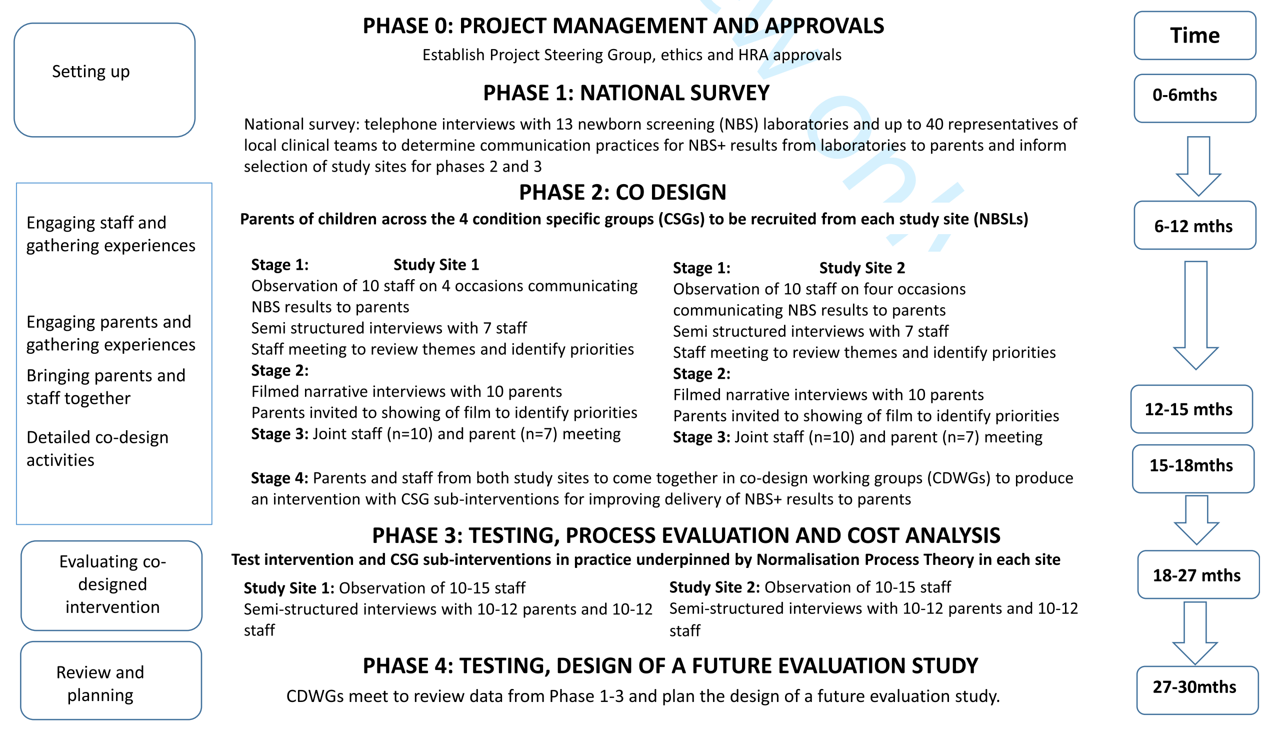

Fig. 1 Study flow chart

For peer review only - http://bmjopen.bmj.com/site/about/guidelines.xhtml

Table 1 Condition-specific groups (CSGs)

Group	Urgency	Conditions
Genetic/metabolic	Urgent—at immediate risk	Medium-chain acyl-CoA dehydrogenase deficiency, maple syrup urine disease, isovaleric aciduria
Genetic/metabolic	Urgent—not at immediate risk	Sickle cell disorder (SCD), cystic fibrosis (CF), phenylketonuria, homocystinuria and glutaric aciduria type 1
Other affected	Important—not at immediate risk	Hypothyroidism
Carriers	Non-urgent	SCD, CF

last 6 months or who have personal experience of receiving a NBS+ result will be excluded.

Recruitment

For phase 1, contact details of Directors of NBS laboratories will be identified through the relevant website[2]. Directors of newborn screening laboratories will be invited to be the Lead Investigator for the study site and will be asked to provide names and contact details of staff within the laboratory who meet the inclusion criteria for the study. These staff members will be contacted via email and invited to participate. Members of relevant clinical teams will be identified through the individual trust websites.

During phases 2 and 3, staff identified in phase 1 as being involved in the communication of positive newborn screening results to parents in the selected study sites will be contacted via email and invited to participate in these phase of the study.

During phases 2 and 3, parents who fit the inclusion criteria will be identified by the relevant clinical nurse specialist. Once eligible parents have been identified, a member of the clinical team (Clinical Nurse Specialist or doctor) will provide the parent with a participant information sheet at their next routine clinic appointment and ask the parents' permission to provide their name and telephone number to a member of the research team. At least 24 h later, a member of the research team will telephone the parents, give them the opportunity to ask questions about the study and ask if they wish to proceed with being involved.

During phase 4, key stakeholders will be identified by the study steering committee using purposive sampling to ensure the four CSGs are represented.

Demographic data

For all parents recruited to the study, data regarding their age, gender and ethnicity will be collected. In addition, they will be asked if the child who has received the NBS+ result is their first child and whether they have any other children. If they have other children, the ages of these children as well as whether or not they also have any long-term medical conditions will also be ascertained.

Consent

Written informed consent will be gathered for all participants for each phase.

Phase 1: National survey

A national survey will be conducted to identify examples of current approaches, and associated resource use, for communication of NBS+ results from all 13 NBS laboratories via clinical teams to parents for each CSG in England. The survey will be informed by the literature and piloted before use in the main study. The survey, comprising closed and open-ended questions, will be conducted using semi-structured telephone interviews. It will identify the ways NBS+ results are communicated from the NBS laboratories to parents via a range of health professionals by collecting data on the following: the mode of communication strategy (face-to-face, letter, telephone, e-mail), the resources involved in each communication strategy, who provides the information and their role, and location (co-located or alternative site) of relevant services for the CSG. The communication pathway currently used will be identified from the point at which the laboratory produces the test result to when the parents are told the definitive result.

Participants

Directors of all 13 NBS laboratories in England will be invited to participate. In addition, up to 40 representative members (10 for each CSG) of local clinical teams (medical consultants, general paediatricians, nurse specialists, health visitors, specialist screening nurses, genetic counsellors) who receive laboratory results and are identified as being involved in communicating positive newborn screening results to parents will be invited as well.

Data analysis

The purpose of data analysis will be to describe and identify approaches currently used to communicate the NBS+ results and identify potential study sites for phase 2 using a predefined sampling framework (Fig. 2). Quantitative

[2] http://newbornscreening.org/site/index.asp

For peer review only - http://bmjopen.bmj.com/site/about/guidelines.xhtml

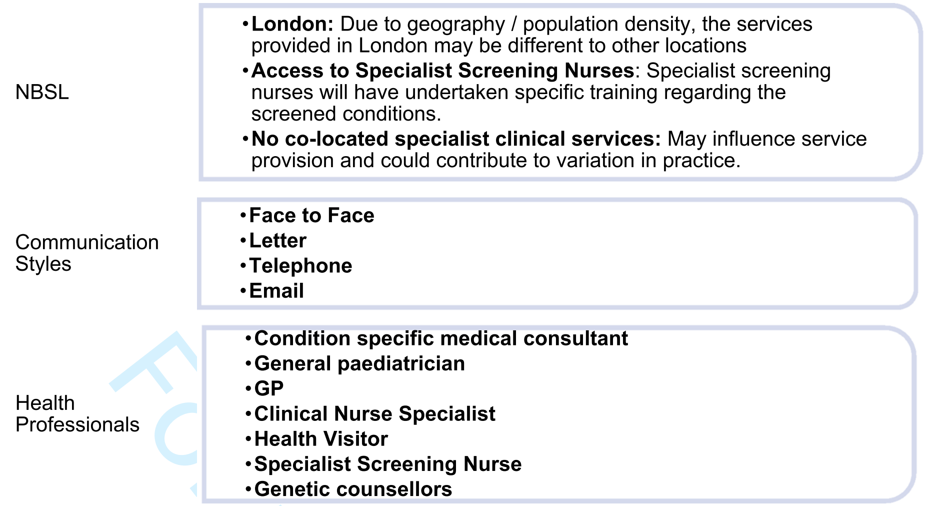

Fig. 2 Exemplar sampling framework: features of the communication process for NBS+ results to parents

data collected from the closed-ended questions will be analysed using descriptive statistics. This might include, for example, the number of people involved for each CSG, the median and range of times each NBS laboratory has been processing NBS results and the median and range of times taken when communicating a NBS+ result to clinical teams. Qualitative data from the open-ended questions will be analysed using content analysis [30]. An inductive approach will be adopted. One interview transcript will be coded separately by two members of the research team (double coded) in order to aid coding comparisons and inform and align code development [31]. A code book will be developed based on these comparisons and subsequent discussions in order to define the codes to be used. This will be an ongoing, iterative process; new codes may be developed during data analysis and the definition of codes refined as analysis progresses [32] Communication pathways for each of the 13 NBS laboratories (unit of analysis) will be described, by combining quantitative and quantitative data. These data will be presented to members of the study steering committee and the lay advisory group who will be involved in the decision regarding which study sites will be used in subsequent study phases. These decisions will be based on data from phase 1 regarding communication strategies currently in use, statistics regarding the number of babies who receive NBS+ results in each of the NBS laboratories on an annual basis [2, 3] and the sampling framework (Fig. 2).

Data collected during this phase will also be used in phase 3 to determine the total cost of existing communication strategies, from the NHS perspective by determining the grade of the person involved in the communication, the time taken and resources used.

Output(s)

The outputs are the following: (i) report describing current communication practices and (ii) selection of relevant study sites for phases 2–3.

Phase 2: Co-design

This phase will consist of implementing the EBCD approach [21, 24] in the two study sites selected in phase 1 and will be guided by the EBCD Toolkit[3]. EBCD is an approach to improving healthcare services that draws on participatory design and user experience to bring about quality improvements in healthcare organisations [23]. EBCD involves focussing on and designing patient/carer experiences rather than just systems and processes [19, 24, 25] and—through a 'co-design' process—enables staff, patients and carers to reflect on their shared experiences of a service and then work together to identify improvement priorities, devise and implement changes, and then jointly reflect on their achievements.

In line with the EBCD approach, this phase will include non-participant observation of staff communicating the NBS+ result to parents. When the relevant member of the clinical team contacts the family to communicate the initial NBS+ result (by whichever methods they normally use, e.g. phone or face-to-face), they will ask the family at the beginning of the interaction, whether a member of the research team may be present. If the family agree, a researcher will observe the clinician communicating the NBS+ result to the family. During the communication, the researcher will not participate in the interaction between the clinician and the family but will take detailed field notes. Semi-structured interviews with staff to explore their experiences of communicating NBS+ to families and then a staff event to review themes that have arisen and identify priorities

For peer review only

going forward will also be undertaken. Alongside this, filmed narrative interviews with parents of children who have received a positive NBS result will be conducted. This will be followed by a parent event where a composite film of the narrative interviews will be viewed and emerging issues and priorities identified. Following the separate staff and parent events, a mixed staff and parent event will be held at each site. These will enable joint priorities for improving delivery of NBS+ results to be developed using issues highlighted in the film and priorities from the separate staff and parent meetings. The joint priorities will guide the four co-design working groups who will meet on three occasions each. During co-design working group meetings, parents and staff will work together to co-design the new interventions based on the jointly identified priorities and following the EBCD toolkit[3].

Participants

Samples sizes for this phase are based on previous successful EBCD studies [20–25]. A purposive sample of 15 staff representing the four CSGs delivering NBS+ results to parents across the two study sites will be the subjects for the non-participant observation. These staff will be interviewed about their experiences and invited to the staff event.

Narrative interviews will be conducted with a purposive sample of 20 parents representing the four CSGs who have received a NBS+ result for their child during the previous 3–12 months. All parents interviewed will be invited to the parent event.

The 15 staff and the 20 parents who have been interviewed will be invited to participate in the joint staff and parent event and the co-design working groups. As per the EBCD toolkit[3], if retention of parents or staff is problematic at this stage, new participants will be recruited for the co-design work.

Data analysis

Data from observation and interviews with staff will be pseudonymised; each participant will be allocated a study code. Data obtained during observation of staff will be analysed using an inductive approach and themes will be generated using a manifest/semantic approach [33] in order to provide a rich description of current practice.

Staff interviews will be audio recorded and transcribed verbatim and will be analysed for themes [33] to inform the joint staff/parent meeting and subsequently the co-design working groups (CDWGs). An inductive approach to data analysis will be used and themes will be generated using a latent approach [33] to provide a deeper understanding of approaches used to communicate positive NBS results to families. The six phases of thematic analysis described by Braun and Clarke [33] will guide data analysis. One interview for each of the CSGs will be coded separately by two members of the research team (double coded) using NVivo software. This will aid coding comparisons and inform and align code development [31]. A code book will be developed based on these comparisons and subsequent discussions in order to define the codes to be used. This will be an ongoing, iterative process; new codes may be developed during data analysis and the definition of codes refined as analysis progresses [32]. Collating codes into potential themes and reviewing and defining the themes will be undertaken jointly by the two members of the research team who generated the initial codes [31, 33]. Themes identified from parent interviews will be made into a 30-min composite film [20–25]. The joint staff/parent meeting will generate the priorities that will be focussed upon by the co-design working groups.

The four CDWGs consisting of parents and staff will work together on proposed solutions for the identified priorities[3]. After each CDWG meeting, ideas generated by the group will be amalgamated and developed into protocols to aid implementation during phase 3. At the next CDWG meeting, the proposed interventions will be presented to the group so they may continue to work together to refine their ideas. The final interventions in the form of protocols will be presented at the final CDWG meetings to determine if any further tweaks are necessary before they are implemented in phase 3 for testing, process evaluation and cost analysis.

Output(s)

The output is the co-designed interventions for the four CSGs developed during the co-designed working group meetings[3].

Phase 3: Testing, process evaluation and cost analysis

It is envisaged that the interventions will involve procedural changes that may include changes to documentation as well as staff training. The former will involve liaison between the research team, clinical teams and Public Health England, via the study support groups. Staff involved in the delivery of NBS+ results in the study sites will be trained to implement the new intervention protocols (developed as a result of the co-designed meetings) for the four CSGs concurrently. A training manual will be developed. Members of the research team will visit each study site and provide clinical teams for the CSGs with two face-to-face training sessions. Follow-up support will be provided including resource packs of information to support the use of the new co-designed interventions in practice, online resources made available to staff via a

[3]https://www.kingsfund.org.uk/projects/ebcd

For peer review only - http://bmjopen.bmj.com/site/about/guidelines.xhtml

study-specific website and remote support via telephone/email. The face-to-face training will include a didactic approach but will also include the use of role play. Staff will be asked to evaluate the training to ensure it has met their needs and identify areas for improvement. Success criteria including the acceptability and feasibility of the co-designed interventions will be defined and monitored on a weekly basis during implementation (Fig. 3).

A parallel process evaluation [18] underpinned by Normalisation Process Theory [26, 27] will be conducted. Non-participant observation of staff delivering NBS+ results to parents and semi-structured interviews with parents and staff will identify healthcare resources required for delivery of the interventions, staff and parental experiences and factors that influence implementation [34, 35]. These qualitative data will also be used to determine suitable outcome measures for a future evaluation study.

An economic analysis will also be conducted. Our analytical approach will be to undertake a cost analysis of the intervention coupled with a feasibility study to plan the economic evaluation that would accompany a definitive evaluation study. The analytical approach that we envisage using in the definitive study would either be a cost-utility analysis or a cost-consequence analysis, and which of these will be most appropriate will be determined during the present feasibility study. The objectives are to (i) calculate the costs of the current and newly co-designed communication strategies (using data collected in phase 3 and phase 1) and (ii) inform the design of a feasibility study for an economic evaluation of options to improve delivery of initial NBS+ results to parents (in phase 4).

To meet the first objective, we will conduct an economic analysis to evaluate the NHS costs of the current and new communication strategies. This will include training costs, staff costs, and costs of consumables (e.g. information booklets). For training costs, we will include costs of the trainer time, training materials, training attendee numbers and time and any other costs associated with training that might be incurred, such as room hire. We will identify resource use for each of these items from training providers and value them using market prices. For staff costs and consumables associated with delivery of each communication strategy, we will produce flow charts for each strategy identifying the main activities and consumables. We will then produce a pro forma for each strategy and ask staff delivering the strategies to record the resources used on this pro forma which will be an electronic document that upon completion can be emailed directly to the study team. We envisage the main cost is likely to be staff time, so therefore, the pro forma will record the amount of time spent on different activities and the staff type and grade performing that activity. Staff time and consumables will be valued using published market prices. The main output will be a pictorial display (flow chart) of each strategy, resource use associated with that strategy and its cost.

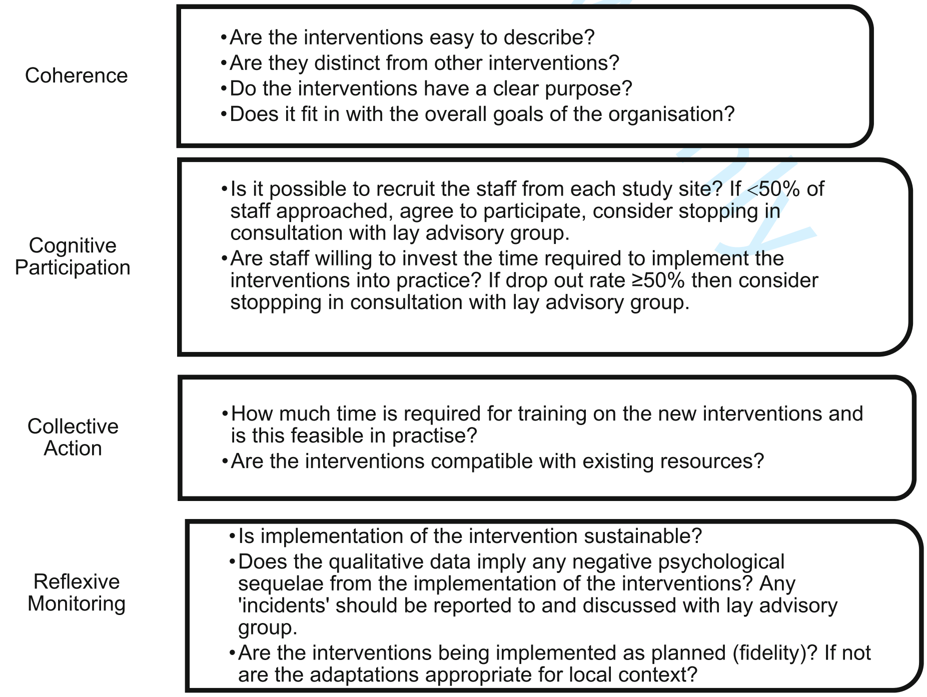

Fig. 3 Success criteria for testing the co-designed interventions in routine practice

For peer review only

For the second objective, we will plan the economic evaluation that would accompany a full evaluation study, identify potential sources of data and how best to collect these. Hence, the aim of the economic analysis in the present study is not to provide a definitive analysis of the costs, cost-effectiveness and budget impact of the planned interventions as that will not be possible until the full evaluation study (assuming this is shown to be feasible).

Participants

Thirty staff involved in delivery of NBS+ results will be trained to implement the co-designed interventions. These staff will be observed delivering NBS+ results to parents using the new co-designed interventions. Twenty parents who have received a NBS+ result in the previous 3–12 months and 20 staff who have communicated a NBS+ result to parents in the previous 12 months will be interviewed. Sample sizes are based on recommendations and findings regarding pilot and feasibility studies [36, 37] to demonstrate feasibility of recruitment, implementation and sustainability of the co-designed interventions in practice.

Data analysis

Qualitative data collected during the observation and semi-structured interviews will be used to identify factors that influence experiences during the delivery of NBS+ results. These data will be compared with the following validated scales: GAD-7 (generalized anxiety disorder), PHQ-9 (Patient Health Questionnaire), the Parenting Stress Index [38], EQ5D[4] (a measure of health-related quality of life) and the ICECAP-A [39] (a measure of capability for the general adult population for use in economic evaluations) to determine where most overlap occurs to inform which outcomes might be most suitable in a future evaluation study.

Data from the non-participant observation will be analysed thematically [33] following the process described in phase 2. An inductive approach to data analysis will be used, and themes will be generated using a manifest and latent approach [33]. These might include the structures (processes) and use of healthcare resources (type and time) required for delivery of the interventions, how parents and staff respond (implementation and mechanism of impact) and how external factors (language barriers, cultural difference) influence implementation of the intervention(s) (context) [34, 35]. All interviews will be audio-recorded and transcribed. A deductive approach to thematic analysis will be used, and themes will be generated using both a manifest and latent approach as per phase 2 [33].

[4]http://www.euroqol.org/home.html

For the economic analysis, we will assess the costs of each communication strategy from an NHS perspective (i.e. calculate the costs to the NHS of delivering that strategy). Resource use data for each strategy will be collected as described above and combined with market prices (unit cost data form published and other sources) to calculate the total costs associated with each strategy.

Output(s)

The outputs are the following: (i) resource use and cost of current communication strategies (using data from phase 1) compared to resource use and costs associated with the co-designed interventions, (ii) acceptability and feasibility of the co-designed interventions based on the success criteria in Fig. 3, and (iii) choice of potential outcomes measures (GAD 7 PHQ 9 PSI [38] EQ5D[4] and ICECAP-A [39]) for use in a future evaluation study.

Phase 4: Design of future evaluation study

A meeting of key stakeholders (NBS co-ordinators, directors of NBS Laboratories, health visitors, midwives, genetic counsellors, parents) will be convened, and the nominal group technique (NGT) [28, 29] used to inform the design of an evaluation study of the co-designed interventions. The NGT was first developed by Delbeq and Van de Ven [40] and consists of a structured meeting consisting of steps during which participants generate, rate, discuss and then rerate a series of items or questions to achieve consensus regarding a given topic [28, 29]. In this study, adaptations to the NGT described in the literature [41–43] will be used during the initial round of idea generation in order to incorporate data collected during phases 1–3 of the study. An outline of the use of the NGT in the study can be seen in Table 2.

Participants

A purposive sample of staff and parents ($n = 10$) involved in phase 2 as well as representatives from relevant charities and members of the research team will be invited.

Data analysis

Qualitative data collated during the NGT will be analysed using thematic analysis [33]. A deductive approach will be employed, and themes will be generated using a manifest/sematic approach in order to stay close to the data. The process described by Braun and Clarke [33] will be followed as described in phase 2, but data will be jointly coded by two members of the research team using NVivo software to ensure reliability of coding [31]. Quantitative data, such as ranking or rating data, will be summarised using descriptive statistics.

Table 2 The modified nominal group technique process

(i) Generating ideas	Members of the group will be asked to individually consider the data that has been presented to them and consider:<br>1. If there is there a need for an evaluation study of the co-designed interventions and if so;<br>2. Ideas for the potential design of an evaluation study of the co-designed interventions.
(ii) Recording ideas	Group members engage in a round-robin feedback session to concisely record each idea (without debate).<br>If it becomes apparent that the consensus is not to proceed to an evaluation study, the NGT will cease at this stage. If it is decided that an evaluation study should be designed based on the data from phases 1–3, member of the group will be asked to share their ideas for the potential design of an evaluation study of the co-designed interventions.
(iii) Clarification	A discussion focussed on clarification of the ideas generated.
(iv) Voting	Individual group members vote privately to rank ideas. The votes will be tallied to identify the ideas that are rated highest by the group as a whole and will then be presented back to the group.
(v) Discussion	A group discussion to provide further clarification of the highest rated ideas for the future evaluation study of the co-designed interventions.
(vi) Re-ranking	Re-ranking of ideas to determine priorities and future plans.

Outcome(s)

The outcomes are the following: (i) need for and design of a future evaluation study which may be condition-dependent and (ii) choice of relevant outcome measures.

Dissemination

We intend to share our study findings on the national NBS websites so that they may be available to relevant health professionals involved in the delivery of the initial positive NBS result. We also plan to share our findings on the websites of the relevant charities and support groups associated with these conditions all of whom have been contacted and provided their endorsement for this study (CF Trust, Sickle Cell Society, British Thyroid Foundation, National Society for Phenylketonuria, Metabolic Support UK). We also aim to share our findings at relevant conferences nationally and internationally. We will submit our findings to high impact, peer-reviewed journals including the NIHR HS&DR journal. Parents involved in the study and those who form the advisory group will also be sent a summary of the research findings.

Discussion

This protocol paper describes a national (England) study to develop co-designed interventions for the communication of NBS+ results to parents.

An important consideration when designing this study was the ethical issues associated with researching such a sensitive topic, i.e. communication of information to parents regarding life-changing/limiting conditions. Asking parents to recount the moment they received their child's positive newborn screening result could be distressing for parents. However, the research team is highly experienced at working with families in this situation and will always proceed with due care and sensitivity. A member of the research team is a Consultant Clinical Psychologist and will be able to assist and advise should a parent become distressed during the interviews. At the end of each interview and parent/staff meeting and CDWG meetings, participants will be debriefed. Parents will also be offered options regarding where they would prefer the interviews to be conducted to minimise intrusion.

This study will lead to the development of co-designed interventions that will meet the needs of parents and health care professionals and will aim to minimise negative sequelae associated with communication of NBS+ results. This study will also involve the calculation and comparison of costs associated with different communication strategies as well as subsequent use of healthcare resources. This will include comparisons between costs of different approaches currently used (from phase 1) and costs of the new, co-designed interventions in terms of grade of staff involved, time taken and resources used (phase 3).

The proposed research may lead to the development of general evidence-based principles for communicating positive screening results for children and breaking bad news. This latter might include conditions that may or may not be life-altering/threatening but nevertheless can be distressing for parents, for example, newborn hearing screening, physical examination of newborn babies including congenital cardiac abnormalities, congenital cataracts, cryptorchidism, developmental dislocation of the hip and findings from screening of children's eyes. It may also be possible to extrapolate findings from the present study for the delivery of bad news to parents for children newly diagnosed with cancer or following diagnosis of chronic conditions such as diabetes or epilepsy.

In order to monitor the success and progress of the study, an independent study steering committee, a project advisory group and a lay advisory group will be convened at the start of the study and will meet in person every 6 months for the duration of the study. The research team will also liaise via telephone conference call monthly for the duration of the study.

For peer review only

The study steering committee will consist of an independent Chair, external stakeholders (such as representatives from Public Health England and relevant charities), relevant methodologists (such as a health economist) and a clinician. The purpose of this committee will be to provide advice on aspects of the study to stakeholders, monitor the progress of the study, ensure the rights, well-being and safety of participants are maintained, ensure appropriate ethical and other approvals are obtained and agree substantial protocol amendments.

Abbreviations

CDWG: Co-design working group; CF: Cystic fibrosis; CSG: Condition-specific group; EBCD: Experience-based co-design; EQ5D: Euro Qol 5D; GAD: Generalised Anxiety Disorder Assessment; ICECAP-A: ICEpop CAPability measure for Adults; NBS: Newborn blood spot screening; NBS+: Newborn blood spot screen positive; NBSP: Newborn blood spot screening programme; NGT: Nominal group technique; PHQ: Patient Health Questionnaire; SCD: Sickle cell disease

Acknowledgements

We thank the British Thyroid Foundation, the Cystic Fibrosis Trust, Metabolic Support UK, The Sickle Cell Society, the Newborn Screening Programme, Public Health England and the parents who gave up their valuable time to develop this protocol.

We would also like to thank Professors Alison Metcalfe and Glenn Robert for their input into the design of this study.

Sponsor

Chris Hull (on behalf of City, University of London), Associate Dean for Research and Enterprise, School of Health Sciences, Northampton Square, City University London, c.c.hull@city.ac.uk

Authors' contributions

JC wrote the first draft of the manuscript. JC led the design of the study and applied for the funding. JC, JB, KS, SM, JF, AS, LM, FU and MB critically reviewed the manuscript. JB, KS, SM, JF, AS, LM, FU and MB participated in the design of the study. All authors read and approved the final manuscript.

Funding

This project was funded by the National Institute for Health Research Health Services and Delivery Research (NIHR HS&DR) Programme (project number 16/52/25). Visit the HS&DR website for more information https://www.journalslibrary.nihr.ac.uk/programmes/hsdr/165225/#/.

The funders provided a scientific critique of the proposed study design, methods of data collection and analysis. The Funders and Sponsor will not be involved in the interpretation of the data and were not involved in writing the manuscript.

Availability of data and materials

Not applicable.

Ethics approval and consent to participate

This study has been approved by the London Stanmore Ethics Committee 17/LO/2102.

All participants in this study will be required to provide written, informed consent.

Consent for publication

Not applicable.

Competing interests

The authors declare that they have no competing interests.

Author details

[1]Centre for Maternal and Child Health Research, City, University of London, London EC1V 0HB, UK. [2]Division of Pharmacy, Diagnostics and Genetics Sheffield Children's NHS Foundation Trust, Sheffield S10 2TH, UK. [3]Paediatric Psychology and Play Services, Great Ormond Street Hospital for Children NHS Foundation Trust, London WC1N 3JH, UK. [4]Division of Health Services Research and Management, City, University of London, London EC1V 0HB, UK. [5]School of Art and Design, Coventry University, Coventry CV1 5FB, UK. [6]Centre of Applied Health Research, University College London, London WC1E 6BT, UK. [7]Centre for Mental Health Research, City, University of London, London EC1V 0HB, UK. [8]Division of Psychology and Mental Health, The University of Manchester, Manchester M13 9PL, UK. [9]Women's and Children's Health, University of Liverpool, Liverpool L69 3BX, UK.

Received: 5 December 2018 Accepted: 8 August 2019
Published online: 04 September 2019

References

1. Public Health England. Newborn blood spot screening: programme handbook. London: Public Health England; 2014.
2. Public Health England. Data collection and performance analysis report newborn blood spot screening in the UK 2015/16. London: Public Health England; 2017.
3. Public Health England. NHS sickle cell and thalassaemia screening programme data report 2015/16: trends and performance analysis. London; 2017.
4. Parker H, Qureshi N, Ulph F, Kai J. Imparting carrier status results detected by universal newborn screening for sickle cell and cystic fibrosis in England: a qualitative study of current practice and policy challenges. BMC Health Serv Res. 2007;7:203.
5. Kai J, Ulph F, Cullinan T, Qureshi N. Communication of carrier status information following universal newborn screening for sickle cell disorders and cystic fibrosis: qualitative study of experience and practice. Health Technol Assess. 2009;13(57):1–82 iii.
6. Ulph F, Cullinan T, Qureshi N, Kai J. Parents' responses to receiving sickle cell or cystic fibrosis carrier results for their child following newborn screening. Eur J Hum Genet. 2015;23(4):459–65.
7. Salm A, Yetter E, Tluczek A. Informing parents about positive newborn screening results: Parents' recommendations. J Child Health Care. 2012;16(4):367–81.
8. Ulph F, Cullinan T, Qureshi N, Kai J. The impact on parents of receiving a carrier result for sickle cell or cystic fibrosis for their child via newborn screening. Eur J Hum Genet. 2014;22(Suppl 1):367.
9. Chudleigh J, Buckingham S, Dignan J, O'Driscoll S, Johnson K, Rees D, et al. Parents' experiences of receiving the initial positive newborn screening (NBS) result for cystic fibrosis and sickle cell disease. J Genet Couns. 2016;25:1215–26.
10. Buchbinder M, Timmermans S. Newborn screening for metabolic disorders: parental perceptions of the initial communication of results. Clin Pediatr (Phila). 2012;51(8):739–44.
11. Rueegg CS, Barben J, Hafen GM, Moeller A, Jurca M, Fingerhut R, et al. Newborn screening for cystic fibrosis - the parent perspective. J Cyst Fibros. 2016;15(4):443–51.
12. Tluczek A, Clark R, McKechnie AC, Brown RL. Factors affecting parent-child relationships one year after positive newborn screening for cystic fibrosis or congenital hypothyroidism. J Dev Behav Pediatr. 2015;36(1):24–34.
13. Kladny B, Williams A, Gupta A, Gettig EA, Krishnamurti L. Genetic counseling following the detection of hemoglobinopathy trait on the newborn screen is well received, improves knowledge, and relieves anxiety. Genet Med. 2011;13(7):658–61.
14. Tluczek A, Orland KM, Cavanagh L. Psychosocial consequences of false-positive newborn screens for cystic fibrosis. Qual Health Res. 2011;21(2):174–86.
15. Public Health England. Press release: newborn babies screened for more rare conditions. London: Public Health England; 2015.
16. Segrin C, Flora J. Family communication. 2nd ed. London: Routledge; 2011.
17. Rolland JS, Williams JK. Toward a biopsychosocial model for 21st-century genetics. Fam Process. 2005;44(1):3–24.
18. Craig P, Dieppe P, Macintyre S, Michie S, Nazareth I, Petticrew M. Developing and evaluating complex interventions: new guidance; 2006.
19. Bate SP, Robert G. Bringing user experience to health care improvement: the concepts, methods and practices of experience-based design. Oxford: Radcliffe Publishing; 2007.
20. Tsianakas V, Robert G, Maben J, Richardson A, Dale C, Wiseman T. Implementing patient centred cancer care: using experience-based co-

For peer review only

For peer review only - http://bmjopen.bmj.com/site/about/guidelines.xhtml

design to improve patient experience in breast and lung cancer services. Support Care Cancer. 2012;20(11):2639–47.
21. Robert G. Participatory action research: using experience-based co-design (EBCD) to improve the quality of health care services. In: Ziebland S, Coulter A, Calabrese J, Locock L, editors. Understanding and using health experiences: improving patient care. Oxford: Oxford University Press; 2013.
22. Locock L, Robert G, Boaz A, Vougioukalou S, Shuldham C, Fielden J, et al. Using a national archive of patient experience narratives to promote local patient-centered quality improvement: an ethnographic process evaluation of 'accelerated' experience-based co-design. J Health Serv Res Policy. 2014;19(4):200–7.
23. Donetto S, Pierri P, Tsianakas V, Robert G. Experience-based co-design and healthcare improvement: realising participatory design in the public sector. Des J. 2015;18(2):227–48.
24. Robert G, Cornwell J, Locock L, Purushotham A, Sturmey G, Gager M. Patients and staff as codesigners of healthcare services. BMJ. 2015;350:g7714.
25. Tsianakas V, Robert G, Richardson A, Verity R, Oakley C, Murrells T, et al. Enhancing the experience of carers in the chemotherapy outpatient setting: an exploratory randomised controlled trial to test impact, acceptability and feasibility of a complex intervention co-designed by carers and staff. Support Care Cancer. 2015;23:3069–80.
26. May CR, Finch T, Ballini L, MacFarlane A, Mair F, Murray E, et al. Evaluating complex interventions and health technologies using normalization process theory: development of a simplified approach and web-enabled toolkit. BMC Health Serv Res. 2011;11:245.
27. Murray E, Treweek S, Pope C, MacFarlane A, Ballini L, Dowrick C, et al. Normalisation process theory: a framework for developing, evaluating and implementing complex interventions. BMC Med. 2010;8:63.
28. Jones J, Hunter D. Qualitative research: consensus methods for medical and health services research. Br Med J (Clin Res Ed). 1995;311:4.
29. Fink A, Kosecoff J, Chassin M, Brook R. Consensus methods: characteristics and guidelines for use. Am J Public Health. 1984;74(9):4.
30. Krippendorff K. Content analysis: an introduction to its methodology. 3rd ed. SAGE: Los Angeles; 2013.
31. Milford C, Kriel Y, Njau I, Nkole T, Gichangi P, Cordero JP, et al. Teamwork in qualitative research: descriptions of a multicountry team approach. Int J Qual Methods. 2017;16:1–10.
32. DeCuir-Gunby JT, Marshall PL, McCulloch AW. Developing and using a codebook for the analysis of interview data: an example from a professional development research project. Field Methods. 2011;23(2):136–55.
33. Braun V, Clarke V. Using thematic analysis in psychology. Qual Res Psychol. 2006;3(2):77–101.
34. Moore G, Audrey S, Barker M, Bond L, Bonell C, Hardeman W, et al. Process evaluation of complex interventions. London: MRC Population Health Science Research Network; 2014.
35. Moore GF, Audrey S, Barker M, Bond L, Bonell C, Hardeman W, et al. Process evaluation of complex interventions: Medical Research Council guidance. BMJ. 2015;350:h1258.
36. Hertzog MA. Considerations in determining sample size for pilot studies. Res Nurs Health. 2008;31(2):180–91.
37. Billingham SA, Whitehead AL, Julious SA. An audit of sample sizes for pilot and feasibility trials being undertaken in the United Kingdom registered in the United Kingdom Clinical Research Network database. BMC Med Res Methodol. 2013;13:104.
38. Abidin RR. Parenting stress index, fourth edition short form (PSI-4 SF). Florida: Psychological Assessment Resources Inc.; 2012.
39. Al-Janabi H, Flynn TN, Coast J. Development of a self-report measure of capability wellbeing for adults: the ICECAP-A. Qual Life Res. 2012;21(1):167–76.
40. Delbeq A, Van de Ven A. A group process model for problem identification and program. J Appl Behav Sci. 1971;7:467–92.
41. Wainwright D, Boichat C, McCracken LM. Using the nominal group technique to engage people with chronic pain in health service development. Int J Health Plann Manag. 2014;29(1):52–69.
42. McMillan SS, King M, Tully MP. How to use the nominal group and Delphi techniques. Int J Clin Pharm. 2016;38(3):655–62.
43. Rankin NM, McGregor D, Butow PN, White K, Phillips JL, Young JM, et al. Adapting the nominal group technique for priority setting of evidence-practice gaps in implementation science. BMC Med Res Methodol. 2016;16(1):110.

Publisher's Note

Springer Nature remains neutral with regard to jurisdictional claims in published maps and institutional affiliations.

Ready to submit your research? Choose BMC and benefit from:

- fast, convenient online submission
- thorough peer review by experienced researchers in your field
- rapid publication on acceptance
- support for research data, including large and complex data types
- gold Open Access which fosters wider collaboration and increased citations
- maximum visibility for your research: over 100M website views per year

At BMC, research is always in progress.

Learn more biomedcentral.com/submissions

BMC

For peer review only

Consolidated criteria for reporting qualitative studies (COREQ): 32-item checklist

Developed from:
Tong A, Sainsbury P, Craig J. Consolidated criteria for reporting qualitative research (COREQ): a 32-item checklist for interviews and focus groups. *International Journal for Quality in Health Care*. 2007. Volume 19, Number 6: pp. 349 – 357

YOU MUST PROVIDE A RESPONSE FOR ALL ITEMS. ENTER N/A IF NOT APPLICABLE

No. Item	Guide questions/description	Reported on Page #
**Domain 1: Research team and reflexivity**		
Personal Characteristics		
1. Inter viewer/facilitator	Which author/s conducted the inter view or focus group?	Section 2.5
2. Credentials	What were the researcher's credentials? E.g. PhD, MD	PhD
3. Occupation	What was their occupation at the time of the study?	Children's Nurse (JC) and Psychologist (HC)
4. Gender	Was the researcher male or female?	Female
5. Experience and training	What experience or training did the researcher have?	MSc Research Methods; extensive clinical experience; interview techniques.
Relationship with participants		
6. Relationship established	Was a relationship established prior to study commencement?	No
7. Participant knowledge of the interviewer	What did the participants know about the researcher? e.g. personal goals, reasons for doing the research	Professional role – Children's nurse, researcher role in this study
8. Interviewer characteristics	What characteristics were reported about the inter viewer/facilitator? e.g. Bias, assumptions, reasons and interests in the research topic	Section 5.0
**Domain 2: study design**		
Theoretical framework		
9. Methodological orientation and Theory	What methodological orientation was stated to underpin the study? e.g. grounded theory, discourse analysis, ethnography, phenomenology, content analysis	Qualitative exploratory design
Participant selection		

For peer review only

10. Sampling	How were participants selected? e.g. purposive, convenience, consecutive, snowball	Purposive
11. Method of approach	How were participants approached? e.g. face-to-face, telephone, mail, email	Email
12. Sample size	How many participants were in the study?	Seventeen
13. Non-participation	How many people refused to participate or dropped out? Reasons?	Three – time constraints / workload demands
Setting		
14. Setting of data collection	Where was the data collected? e.g. home, clinic, workplace	Workplace
15. Presence of non-participants	Was anyone else present besides the participants and researchers?	No
16. Description of sample	What are the important characteristics of the sample? e.g. demographic data, date	Staff involved in communicating the initial positive NBS result in the preceding 6 months.
Data collection		
17. Interview guide	Were questions, prompts, guides provided by the authors? Was it pilot tested?	Yes
18. Repeat interviews	Were repeat inter views carried out? If yes, how many?	N/A
19. Audio/visual recording	Did the research use audio or visual recording to collect the data?	Audio recording
20. Field notes	Were field notes made during and/or after the inter view or focus group?	Yes
21. Duration	What was the duration of the inter views or focus group?	37 minutes (range 19-58 minutes)
22. Data saturation	Was data saturation discussed?	Yes
23. Transcripts returned	Were transcripts returned to participants for comment and/or correction?	No but a staff meeting was held after all interviews had been conducted as per the protocol
**Domain 3: analysis and findings**		
Data analysis		
24. Number of data coders	How many data coders coded the data?	Two
25. Description of the coding tree	Did authors provide a description of the coding tree?	Yes
26. Derivation of themes	Were themes identified in advance or derived from the data?	Derived from the data
27. Software	What software, if applicable, was used to manage the data?	N/A
28. Participant checking	Did participants provide feedback on the findings?	Yes

For peer review only

Reporting		
29. Quotations presented	Were participant quotations presented to illustrate the themes/findings? Was each quotation identified? e.g. participant number	Results section
30. Data and findings consistent	Was there consistency between the data presented and the findings?	Yes
31. Clarity of major themes	Were major themes clearly presented in the findings?	Results
32. Clarity of minor themes	Is there a description of diverse cases or discussion of minor themes?	Discussion

Once you have completed this checklist, please save a copy and upload it as part of your submission. When requested to do so as part of the upload process, please select the file type: *Checklist*. You will NOT be able to proceed with submission unless the checklist has been uploaded. Please DO NOT include this checklist as part of the main manuscript document. It must be uploaded as a separate file.

For peer review only

BMJ Open

A qualitative exploration of health professionals' experiences of communicating positive newborn bloodspot screening results for nine conditions in England

Journal:	*BMJ Open*
Manuscript ID	bmjopen-2020-037081.R1
Article Type:	Original research
Date Submitted by the Author:	30-Apr-2020
Complete List of Authors:	Chudleigh, Jane; City University, School of Health Sciences<br>Chinnery, Holly; St Mary's University Twickenham<br>Bonham, Jim; Sheffield Children's NHS Foundation Trust<br>Olander, Ellinor; City University London<br>Moody, Louise; Coventry University<br>Simpson, Alan; King's College London,<br>Morris, Stephen; University of Cambridge School of Clinical Medicine, Primary Care Unit<br>Ulph, Fiona; University of Manchester<br>Bryon, Mandy; Great Ormond Street Hospital For Children NHS Foundation Trust<br>Southern, Kevin; Institute of Child Health, Paediatrics
**Primary Subject Heading**:	Communication
Secondary Subject Heading:	Paediatrics
Keywords:	GENETICS, PAEDIATRICS, Cystic fibrosis < THORACIC MEDICINE, HAEMATOLOGY, Other metabolic, e.g. iron, porphyria < DIABETES & ENDOCRINOLOGY

SCHOLARONE™
Manuscripts

I, the Submitting Author has the right to grant and does grant on behalf of all authors of the Work (as defined in the below author licence), an exclusive licence and/or a non-exclusive licence for contributions from authors who are: i) UK Crown employees; ii) where BMJ has agreed a CC-BY licence shall apply, and/or iii) in accordance with the terms applicable for US Federal Government officers or employees acting as part of their official duties; on a worldwide, perpetual, irrevocable, royalty-free basis to BMJ Publishing Group Ltd ("BMJ") its licensees and where the relevant Journal is co-owned by BMJ to the co-owners of the Journal, to publish the Work in this journal and any other BMJ products and to exploit all rights, as set out in our licence.

The Submitting Author accepts and understands that any supply made under these terms is made by BMJ to the Submitting Author unless you are acting as an employee on behalf of your employer or a postgraduate student of an affiliated institution which is paying any applicable article publishing charge ("APC") for Open Access articles. Where the Submitting Author wishes to make the Work available on an Open Access basis (and intends to pay the relevant APC), the terms of reuse of such Open Access shall be governed by a Creative Commons licence – details of these licences and which Creative Commons licence will apply to this Work are set out in our licence referred to above.

Other than as permitted in any relevant BMJ Author's Self Archiving Policies, I confirm this Work has not been accepted for publication elsewhere, is not being considered for publication elsewhere and does not duplicate material already published. I confirm all authors consent to publication of this Work and authorise the granting of this licence.

For peer review only

TITLE PAGE

A qualitative exploration of health professionals' experiences of communicating positive newborn bloodspot screening results for nine conditions in England

Jane Chudleigh[1]*, Holly Chinnery[2], Jim R Bonham[3], Ellinor K Olander[1], Louise Moody[4], Alan Simpson[5], Stephen Morris[6], Fiona Ulph[7], Mandy Bryon[8], Kevin W Southern [9]

*corresponding author

[1]Centre for Maternal and Child Health Research, City, University of London, EC1V 0HB, UK;

[2]Faculty of Sports, Health and Applied Science, St Mary's University Twickenham, TW1 4SX

[3]Division of Pharmacy, Diagnostics and Genetics Sheffield Children's NHS Foundation Trust, S10 2TH, UK;

4 Centre for Arts, Memory and Communities, Coventry University, CV1 5FB, UK;

[5] Florence Nightingale Faculty of Nursing, Midwifery & Palliative Care, King's College London SE1 8WA, UK; [6] University of Cambridge School of Clinical Medicine, CB2 0SP, UK; [7]Division of Psychology and Mental Health, The University of Manchester, M13 9PL, UK, [8]Paediatric Psychology and Play Services, Great Ormond Street Hospital for Children NHS Foundation Trust, WC1N 3JH, UK, [9] Women's and Children's Health, University of Liverpool, L69 3BX, UK

j.chudleigh@city.ac.uk; holly.chinnery@stmarys.ac.uk; jim.bonham@sch.nhs.uk; Ellinor.Olander@city.ac.uk; l.moody@coventry.ac.uk; alan.simpson@kcl.ac.uk; sm2428@medschl.cam.ac.uk; fiona.ulph@manchester.ac.uk; mandy.bryon@gosh.nhs.uk; kwsouth@liv.ac.uk

For peer review only - http://bmjopen.bmj.com/site/about/guidelines.xhtml

For peer review only

ABSTRACT

Objective: To explore health professionals experiences of communicating positive NBS results, highlight differences, share good practice and make recommendations for future research.

Design: Qualitative exploratory design was employed using semi-structured interviews

Setting: Three National Health Service provider organisations in England

Participants: Seventeen health professionals involved in communicating positive newborn bloodspot screening results to parents for all nine conditions currently included in the newborn bloodspot screening programme in England.

Results: Findings indicated variation in approaches to communicating positive newborn bloodspot screening results to parents, largely influenced by resources available and the lack of clear guidance. Health professionals emphasised the importance of communicating results to families in a way that is sensitive to their needs. However, many challenges hindered communication including logistical considerations, difficulty contacting the family and other health professionals, language barriers and parental reactions as well as resource considerations; training, and time.

Conclusion: Health professionals invest a lot of time and energy trying to ensure communication of positive newborn bloodspot screening results to families is done well. However, there continues to be great variation in the way these results are communicated to parents and this is largely influenced by resources available but also the lack of concrete guidance. How best to support health professionals undertaking this challenging and emotive task requires further exploration. We recommend evaluation of a more cohesive approach that meets the needs of parents and staff while being sensitive to the subtleties of each condition.

Trial registration: ISRCTN 15330120

Key words: Newborn bloodspot screening, communication

Word count: 5935

For peer review only - http://bmjopen.bmj.com/site/about/guidelines.xhtml

Strengths and limitations of this study

- Health professionals involved in the present study were employed in three different NHS organisations, increasing transferability of the findings and were very experienced, supporting families for on average 10 years
- Participants were recruited via email; those with a pre-existing interest in this topic may have been more likely to self-select into the study. These people may communicate results differently than providers who did not participate in the study
- Health professionals were recruited from clinical teams involved in managing all nine conditions currently included in the NBS programme in England; previous work has mainly focussed on cystic fibrosis and sickle cell disease
- The study design, data collection and analysis were influenced by members of the patient and public involvement advisory group and relevant charities

For peer review only

For peer review only - http://bmjopen.bmj.com/site/about/guidelines.xhtml

INTRODUCTION

Newborn bloodspot screening (NBS) seeks to identify pre-symptomatic babies that are affected by genetic or congenital conditions[1]; early diagnosis leads to better health outcomes for the child[1 2]. In January 2015, NBS in England and Wales expanded and now covers nine conditions: sickle cell disease (SCD); cystic fibrosis (CF); phenylketonuria (PKU); homocystinuria (HCU); glutaric aciduria type 1 (GA1) medium chain acyl CoA dehydrogenase deficiency (MCADD), maple syrup urine disease (MSUD), isovaleric aciduria (IVA) and Congenital Hypothyroidism (CHT).

In the UK, parents are given information prior to and after birth and are asked to provide informed consent for their baby to take part in the NBS Programme. NBS involves a small amount of blood being taken from the baby's heel on day 5 of life. Each year, around 10,000 parents[1] of babies born in the UK are given a positive NBS result. This happens around 2-8 weeks after birth, depending on the condition[3 4]. Parents are informed of the NBS result, normally on the same day it becomes available, and are asked to bring their baby to a particular hospital for diagnostic testing within a given timeframe depending on the suspected condition.

National Guidance regarding the content and mode of communication between health professionals (HPs) and parents is generic and vague[1 5]. Communication of positive NBS results is often delivered by specialist nurses and is a subtle and skilful task which demands thought, preparation and evidence to minimise potentially harmful negative sequela[6-10]. However, training for this role is challenging due to the fact that for many of conditions, a positive results occurs relatively infrequently and may be communicated b several members of the appropriate team people.

There is evidence of variations both nationally and internationally with regard to the approaches used to communicate positive NBS results particularly in relation to CF and SCD[10-13]. These variations include the different approaches used to communicate the results (such as in person during a home visit or by letter)[11 14], the choice of person or profession to deliver the result and the content of the communication[15-19].

---

[1] The term 'parents' refers to parents, carers or guardians.

For peer review only - http://bmjopen.bmj.com/site/about/guidelines.xhtml

The findings of Kai et al's study[14] informed the development of the current national guidelines for the communication process in the NBS programme[1 5], which recommend face-to-face communication by an appropriately trained HP. Despite these guidelines, a study reporting the findings from 67 interviews with parents about their experience of receiving CF or SCD carrier results following NBS indicated that disparity continues to exist regarding how the guidelines are implemented in practice[7]. The findings also indicated variability in the content and the way the result was communicated which led to increased parental anxiety and distress; the perceived lack of knowledge of the person communicating the result was potentially more distressing than the positive finding [7]. This highlights the importance of understanding varied practice in order to identify, share and recommend good practice.

The purpose of this work was to explore HPs' experiences of communicating positive NBS results to highlight differences, share good practice and make recommendations for future research. Experiences of parents are reported elsewhere[20].

METHODS

A qualitative exploratory design was employed using semi-structured interviews as part of a large ongoing programme of work[21] . The study was approved by the London Stanmore ethics committee (17/LO/2102).

Setting

Study sites consisted of three National Health Service provider organisations in England that process comparable numbers of positive NBS reports annually for each of the nine conditions currently included in the NBS Programme. These consisted of two in Greater London (served by one NBS laboratory processing 128 positive NBS results in 2017/18) and one in the West Midlands (whose NBS laboratory processed 129 positive NBS results in 2017/18).

Patient and public involvement

Patient and public involvement (PPI) was instrumental in the design and conduct of this work. Parents of babies who had received a positive NBS screening result for one of the nine screened conditions formed a PPI advisory group who met prior to, during and following data collection. Their suggestions were incorporated into the study design, the data collection tools and the data analysis and presentation. In addition, views of representatives from charities for

For peer review only - http://bmjopen.bmj.com/site/about/guidelines.xhtml

the screened conditions including Metabolic Support UK, the British Thyroid Foundation, the CF Trust and the Sickle Cell Society were also sought.

Inclusion and Exclusion Criteria

HPs involved in communicating positive NBS results in the previous 6 months were invited to take part in the study. HPs who had not been involved in communicating positive NBS results in the last 6 months or who had personal experience of receiving a positive NBS result were excluded.

Recruitment

A purposive/convenience sampling approach was employed. Members of relevant clinical teams (medical consultants; general paediatricians; nurse specialists; specialist screening nurses) were initially identified through individual Trust websites and contacted via email and invited to participate. If no response was received, a follow up email was sent after one week. Identified HPs were asked if there were any other members of the clinical teams that the research team should contact to ensure views were representative. All potential participants were given the choice to participate or not and were reminded of their right to withdraw from the study at any time. Written informed consent was obtained from all participants ahead of the interview.

Data Collection

Semi-structured, face-to-face interviews comprising closed and open-ended questions were conducted by JC and HC from September 2018 to March 2019, to explore HPs experiences of communicating positive NBS to families. Interviews took place at the participants' place of work in a location and at a time of their choice. Topics covered included how HPs manage communication, examples of situations when it has gone well or not as well as expected and the possible reasons for this. We also explored how participants felt during the experience and asked for any suggested changes to practice. Interviews were audio recorded and transcribed verbatim.

Data Analysis

For peer review only - http://bmjopen.bmj.com/site/about/guidelines.xhtml

For peer review only

Interviews were analysed for themes[22]. An inductive approach to data analysis was used and themes generated using a latent approach[22] to provide a deeper understanding of approaches used to communicate positive NBS results to families. The six phases of thematic analysis described by Braun and Clarke[22] guided data analysis.

Two members of the research team (JC and HC) coded one interview transcript separately. These codes were then compared to inform and align code development[23] and a code book was developed. A further four transcripts were then coded separately by the same two members of the research team using the code book. These separately coded transcripts were then compared; inter-coder reliability was 84%. Following this, the same two members of the research team coded the remainder of the transcripts using the code book. Once this initial coding had been completed, all data for each code were compared to ensure consistency in coding and to enable the codes to be collapsed into themes. All quotes for each theme were collated to inform theme development. This was an ongoing, iterative process; new codes were developed and the definition of codes refined as analysis progressed.[24]

Positionality and reflexivity

Members of the study team (JC, JRB, LM, FU, MB, KWS) have been involved in or continue to undertake a variety of roles and activities associated with the NBS Programme in the UK. It is acknowledged that this could have led to potential bias during data collection and analysis. However, this was balanced by other members of the research team who had previously had minimal involvement in NBS (HC, EKO, AS, SM). Data collection and analysis was mainly undertaken by JC and HC who fall within both camps. Neither JC nor HC were employed in the same organisations where data collection was undertaken.

RESULTS

In total, 20 HPs were emailed and invited to participate. Two HPs did not respond to the invitation and one HP did not communicate the initial positive screening result and was therefore ineligible. Therefore, 16 face to face interviews were conducted with 17 HPs (two requested to be interviewed together); eight were from the West Midlands and the remaining nine were split across the two Greater London Trusts. Participants with experience of all the nine screened conditions were included. Interviews lasted on average 37 minutes (range 19-58 minutes). The sample consisted of 8 medical consultants, 1 medical registrar, 7 nurse

For peer review only - http://bmjopen.bmj.com/site/about/guidelines.xhtml

specialists / advanced nurse practitioners and 1 screening nurse. Length of experience with newborn screening ranged from 2-38 years (median 8 years)

Five themes were identified; communication between HPs, process of communicating with the family, parent and family centred care, availability of resources and challenges to effective communication. Illustrative quotations are used to support the themes.

Communication between HPs

HPs reported a range of communication approaches to ensure sufficient information was available to them prior to communicating with families. This started with the laboratory communicating the result to the relevant clinical team in a variety of ways. These included a letter, normally via email or a telephone call followed by an email or a personal visit from a member of the screening laboratory to the clinical nurse specialist, the screening nurse, the on-call consultant or the named consultant depending on the condition, local resources and agreements.

"So we tend to find out from the newborn screening nurses. So, they'll be alerted by the labs and then they would give us [the physician] a call"[SS1P202]

The written initial communication consisted of a pro forma which was often developed locally and may or may not have been accompanied by a copy of the NBS card.

"…generally, we get a pro forma from the screening lab that's slightly different I think depending on the screening lab."[SS2P206]
"We actually started asking for the card."[SS2P201&02]

Receiving a copy of the NBS card was viewed favourably as it enabled HPs to check referral information and parental contact details if this were found to be ambiguous in any way.

Often, this would initiate a two pronged approach where HPs would commence gathering additional information about the child and family from health visitors (registered nurses or midwives who have undertaken additional training and work mainly with children from birth

For peer review only - http://bmjopen.bmj.com/site/about/guidelines.xhtml

to 5 years and their families), midwives and/or primary care physicians before contacting the family.

"Sometimes, it is good to know, the family dynamics, social care issues etc. from somebody [the health visitor or midwife] who's already involved with the family". [SS1P203]

However, clinicians sometimes found it challenging to make contact with health visitors and/or midwives to gather additional information about the family.

"...quite often we are leaving messages to ask them [the health visitor or midwife] to call us back and quite often we are receiving those calls back, after we've already visited the family." [SS1P203]

Simultaneously, members of the multi professional team would also be contacted such as the physiotherapist, dietician or pharmacist (depending on the condition) to inform them that the child would be attending the hospital either the same or the following day.

"The CF nurse specialist would let other members of the team know, so the physio and dietician know that there was a new baby, positive screen so that they could be on standby, but not necessarily to see the family."[SS1P201]

Similarly, if the plan was to see the child in a local hospital rather than in a tertiary care centre, similar communication would happen between the specialist centre and local clinical teams.

Process of communicating with the family

The initial contact with the family was undertaken in a variety of ways by different members of the clinical team. This included, face to face contact in the family's home, via telephone, text message or letter from the screening nurse, relevant clinical nurse specialist or medical consultant. Some respondents commented that they felt the person who told the family should not be a member of the specialist clinical team who would go on to care for the child and they felt this may taint any ongoing relationship. Others felt it was important that the person who gave the initial positive NBS result should be part of the child's clinical team to start building

For peer review only - http://bmjopen.bmj.com/site/about/guidelines.xhtml

familiarity and continuity of care or that there should be someone present who is known to the family.

"So the ideal is that either the midwife or the health visitor can come on the visit so that somebody in the family already knows." [SS1P203]

Views regarding how the initial communication with the family was conducted varied. Some clinicians felt it should be face-to face as it allowed the clinician to gather information about the family that would help to inform the follow-up visit at the hospital with the clinical team.

"You just don't know what's going on in the home environment and you've, sort of, been and witnessed it for yourself and it just gives you a good insight into the family dynamics or what's going on or what support mechanism are in place. You can't get that over the telephone." [SS1P202].

Others felt that a telephone call would be more appropriate to ensure families were told as soon as possible while some felt that a text message asking the family to call them back had worked really well and that a home visit may be quite intrusive. This suggests that sometimes, the approach to communication may not be steered wholly by the needs of the family but sometimes the experience of the person communicating the result.

"You can't really get rid of anyone in someone's house, can you? …You'd be a bit like, 'Right, go now from my house. Get out of my house'...how scary must it be for somebody to turn up on your doorstep"[SS2P201&02]

Regardless of who or how the initial communication took place, all respondents acknowledged the importance of 'getting it right'. Respondents felt it was important the person giving the positive screening result to the family was knowledgeable about the condition they would be discussing with the family. Indeed, many felt this was more important than how the information was delivered and could influence perceptions of ongoing care.

"I think the most important thing, in my opinion, is that the person giving that information, the first time, needs to be someone that can answer some questions." [SS1P207]

For peer review only - http://bmjopen.bmj.com/site/about/guidelines.xhtml

"I think that first telephone call and the first time you see them is absolutely critical…I think the family's views of what's going to come next will be completely modified by how it's done and their confidence in the service." [SS2P206]

However, it was also acknowledged that although various guidelines and protocols existed for the laboratory staff when processing the NBS card and for treatment and management once the child had been diagnosed, there was a paucity of guidance regarding communication of the initial positive NBS result.

"We've got all the protocols around timelines and KPIs etc. but the one bit we don't have anything concrete about is who breaks the news, what level of training or experience they need to have before they do it and what the expectation is of what they should cover in that visit." [SS1P203]

Consequently, the content of the initial communication also varied considerably. Some clinicians spoke about having a template they followed which helped them to ensure they imparted all the information required during what could sometimes be a very emotive interaction. Most agreed that they would try to keep the information about the suspected condition during the initial communication quite brief due to the fact it is a screening result and therefore it would not be appropriate to give too much information about a condition that had not been confirmed. In addition, families were often perceived as being unable to absorb the information very well due to the shock of an unexpected result. Finally, clinicians knew that when families are seen the same or following day, they would receive a lot more information and therefore were reluctant to overload them with information during the first contact.

"So we always have said, 'Screening will be rechecked. The bloods will be redone.'" [SS1P203]

"We don't usually give them much information, they're [the family] usually really upset." [SS2P201&02]

For peer review only - http://bmjopen.bmj.com/site/about/guidelines.xhtml

However, HPs did recognise the importance of signposting families to additional information sources such as charity websites and the National NBS website following the initial communication and gave examples of excellent practice.

"We always take out suspected leaflets. They always get a copy of something to read. We take out a map for the hospital…so they know where to go to in that building….We leave them a letter that confirms what the screening result is and what that might mean for the baby. On the top of that letter, it has our mobile number and office number to give them permission to ring us if they were at all worrying."[SS1P203]

Parent and family centred care

The importance of having a parent and family-centred approach when communicating the positive NBS result was emphasised by all clinicians and was considered to be an example of excellent practice. HPs spoke about the importance of the content of the initial communication. This was subdivided into a beginning, namely, remembering to congratulate parents on the birth of their child. This was followed by a section focussed on tailoring the quantity and level of information given depending on the parental response, not giving too much information or overwhelming parents, being honest and providing emotional support and reassurance where needed.

"So, you just have to judge how much information to give them and how best to support them because if parents are crying and upset about the diagnosis, you obviously have to support them in a different way to other parents." [SS1P205]

Finally, ending with a positive message, giving parents' time and making sure the family know that the baby's mum should be encouraged to bring a support person to the first visit. Also, the importance of treating each family as individuals and acknowledging the enormity of the task they are undertaking.

"I think it's easy, particularly if you're tired or something, to appear a bit more routine, run of the mill, whereas, obviously, it's a really big thing. This is their child, and this is an important piece of information, and it matters hugely to them. You have to try and reflect that

For peer review only - http://bmjopen.bmj.com/site/about/guidelines.xhtml

in how you talk to them, rather than them just being another parent of another sickle baby, which is the danger."[SS3P206]

Some clinicians said they endeavour to find out how much parents already know for instance if they have another child with the condition or if they have been searching the internet before embarking on any explanations. Also, gauging how much families want to know as well as how much families are actually absorbing and when it is time to stop.

"…there are some families that come in that want to know every single detail about everything and you try and be systematic in how you deliver that information. There are other parents that you can tell, although you're trying to tell them things, they are not really taking it on board." [SS1P205]

Finally, HPs recognised the importance of supporting the whole family and therefore not making it the responsibility of the parents to share information or educate other family members.

"We're there to support whoever. The home visit follow-up that I do, I say, 'All are welcome, you know, if granny and granddad want to be there, if they've got questions, I'm happy to speak with them.' Very often, grandparents, aunts and uncles are there, you know. I've been to one and people have been there on mass, but that's their family support network and if that's usually there in place, we don't exclude anyone. I'm happy to talk to any family member." [SS1P202]

For peer review only

Availability of resources

HPs at all levels and for all conditions acknowledged a lack of training and related competencies in terms of breaking bad news to families. This resulted in screening nurses in particular developing their own training programme to address this deficit.

"…we've had to develop some competencies….but because it's so unique and there aren't other nurses funded through a lab post, that there aren't competencies around." [SS1P203]

Resources available at different hospitals also influenced communication of the initial positive NBS result. Therefore, even if clinicians felt that offering to do a home visit to

For peer review only - http://bmjopen.bmj.com/site/about/guidelines.xhtml

deliver the initial positive NBS result would be beneficial, the need to prioritise resources often meant this would not be an option.

"…we don't have the capacity [to do home visits]….I think it will be good if we can do that, but we definitely don't have the capacity for that." [SS2P203]

One hospital had screening nurses and part of their role was to deliver the initial positive NBS result to families, usually face-to-face in their home. In the other two hospitals, this resource was not available and therefore the responsibility of delivering the initial positive NBS result to families stayed with the relevant clinical team. Even though HPs who were able to, felt very positive about the ability to offer home visits, these were resource intensive particularly when compared to the option to contact parents via telephone.

"Probably around 40 minutes is a quick visit. The longest visit I have been there [excluding travel] is probably about three hours."[SS1P203]

The ability to offer timely follow-up appointments with the clinical team both due to clinical need and also to alleviate parental anxiety was also viewed as important. However, many respondents discussed the availability of resources as a potential barrier to this and this seemed particularly evident for babies with SCD.

"It can be a week if we've got a slot, or it can be anything up to four to five weeks….I think it is a very stressful time and some families find that too long." [SS1P209]

For other conditions, clinical need meant that families were seen almost immediately by the clinical team after they had been given the initial positive NBS result.

"So, it's all clinically indicated….the rest of the metabolic conditions will be seen the same day." [SS1P203]

Although ensuring families are seen quickly after being told their child has a positive NBS result was seen as important by the clinical team, this also posed problems on occasions in terms of the financial burden for families needing to travel to the hospital at short notice. Currently, there is no budget to prospectively pay for families' travel expenses to attend

For peer review only - http://bmjopen.bmj.com/site/about/guidelines.xhtml

clinic appointments which means that families are expected to meet these costs in the first instance. Although it may be possible, in certain circumstances, to apply to have these reimbursed at a later date, this does not help families who struggle to pay these costs up front.

"…but we have had families who, it has happened where a family said, 'I just don't think I have the money. I can't afford to come' and saying, 'We'll pay you back' doesn't help and that is difficult" [SS2P206].

Some clinicians also felt that the setting within which the family met the clinical team was important in terms of first impressions.

"This room was designed that it hasn't got a computer or a phone, unlike our other consultation rooms. So, it's a quiet space to deliver the news to the family." [SS1P202]

Availability of resources for diagnostic testing was also viewed as potentially problematic.

"…when we started, the labs were kitted out, such that they would be able to offer a sweat test and a result the same day, any day….because of cuts…they're much less flexible in terms of what they can offer." [SS1P201]

Therefore, although HPs felt very strongly about offering a parent-centred approach to communicating positive NBS results to parents, availability of training, staff and physical space could act as a barrier.

Other concerns related to practical and resource considerations included adding new conditions to the NBS Programme and changes in geographical areas covered by screening laboratories particularly in relation to inherited metabolic diseases and CHT.

"…they [babies with CHT] are dealt with by fifteen different centres…and I think that causes the labs quite a lot of problems. The labs would much prefer fewer centre." [SS2P207]

Challenges to effective communication

For peer review only - http://bmjopen.bmj.com/site/about/guidelines.xhtml

HPs in the current study were asked about their experience of communicating positive NBS result to families. Several staff commented on the personal and emotional impact of this aspect of their role.

"Some can be pretty traumatic and you just feel like you've destroyed their world." [SS1P202]

"It's very emotional for me sometimes, very, very emotional…the mother, a new born, holding a new born baby crying…based on the result you gave them…you wish you could change the result for them." [SS3P211]

Despite this, no formal mechanisms were in place to support HPs. However, all reported that they had developed their own support mechanisms within their teams.

"…everyone's very used to doing it, so everyone completely gets it and completely understands….you have a bit of a debrief with the consultant who's seeing the family." [SS3P202]

HPs also struggled to make contact with parents for a variety of reasons including, the contact details on the NBS card being illegible, parents having moved or staying with relatives after the NBS sample was taken and parents not answering their phones.

" You fill in clearly….not flipping scribbled…the name of the person, a first number, a second number of a partner or someone like that…even though we had a picture of the card, they hadn't filled in any of the details" [SS2P201&02]

"It's not being able to get in contact with people, because they're not picking up on a random London number…The amount of junk calls we all get nowadays, these people are just not picking up the phone." [SS2P201&02]

Once parents had been contacted, other challenges arose related to parental attributes. A common theme related to communicating with parents who did not speak English as a first language. This was not always apparent until after the clinician had attempted to make contact with the parent. This led to parents not understanding what was being communicated,

For peer review only - http://bmjopen.bmj.com/site/about/guidelines.xhtml

the seriousness of what was being communicated or simply not engaging in the conversation due to lack of understanding.

"If English isn't the first language, I certainly find it much more difficult to reassure, and try to be empathetic because you're worried about just getting the basic understanding across." [SS2P206]

Managing families who do not believe the diagnosis either because their child has no signs or symptoms or due to religious or cultural beliefs was also difficult. Such beliefs may also affect engagement with recommended medication and/or treatments. This was particularly evident for those families with a child with SCD.

"They just wouldn't accept that they'd got sickle, because they looked well." [SS1210]

"...so you get a few that either don't believe the diagnosis or their cultural beliefs are that God will mean that they don't have to do anything medical that we suggest." [SS1P205]

Clinicians commented that this often led to patients not attending clinical appointments which also posed challenges in terms of monitoring.

Oher challenges related directly to the NBS Programme. The NBS Programme is designed to identify babies with a higher chance of being affected by one of the screened conditions, often before they are symptomatic, so they can start treatment as soon as possible if needed, and therefore improve outcomes. However, the need to inform parents that their baby has a positive NBS result for a potentially life changing and/or life limiting condition when the baby is asymptomatic was challenging.

"You come to see the doctor because your child is unwell, whereas, this is the opposite, in that, you've got a child who appears perfectly healthy, and you're telling them that they're unwell." [SS1P207]

DISCUSSION

For peer review only - http://bmjopen.bmj.com/site/about/guidelines.xhtml

This study furthers our understanding of HPs' experiences of communicating positive NBS results to families. It is clear that staff involved in communication of positive NBS results are passionate about making sure that although the message is distressing for parents, it is done well. Variation in communication practice continue to exist and is influenced by many factors including resources available but also the lack of clear guidance. This impacted on the methods used to communicate positive NBS results but also the content of the communication to parents. This is supported by previous research which has been conducted both nationally and internationally[7 10 11 13] and suggests that further guidance may be needed to ensure a more cohesive approach which meets the needs of parents' and staff while being sensitive to the subtleties of each condition. However, the issue of finite resources and the need to prioritise these also needs careful consideration.

An overarching message from the HPs involved in the present study was the desire to ensure communicating positive NBS result to families is parent and family-centred. Once HPs were aware of a positive NBS result, they spent a great deal of time 'setting the scene' by gathering information from various sources in preparation for speaking with the family and organising the follow-up appointment with the clinical team. Often this important and necessary work would be time consuming and labour intensive in terms of identifying individuals who either obtained the original sample (midwives) or might have additional information about family dynamics (health visitor) and may be hindered by poor completion or lack of information on the NBS card.

Current guidance[1 5] does not explicitly state who is the 'right' person to communicate a positive NBS result to families or what training or qualifications they should have and specific training to undertake this role is not available. While this does allow flexibility in terms of resources, it can lead to disparity in terms of parental experience of receiving the NBS result. There is no specific guidance regarding exactly what information should be shared with parents during the initial communication[5]. While some HPs alluded to using an informal checklist, this was not universal and therefore may not lead to consistency between clinicians. However, clinicians did recognise the importance of the person imparting the result, having adequate condition specific knowledge, this is consistent with previous research[15 16 19]. These are common problems that have been highlighted both nationally and internationally.[13] As these are not dependent on specific healthcare systems *per se*, the

For peer review only - http://bmjopen.bmj.com/site/about/guidelines.xhtml

findings of the present study could also be extrapolated to screening programmes in other countries.

Clinicians experienced many challenges which hindered the communication of positive NBS results to families. This often stemmed from inadequate information on the NBS card but also parental reactions which could hinder effective communication. When parents are told the NBS result, their baby is often pre-symptomatic as this is one of the purposes of the NBS Programme which means the result is often unexpected.[10 17 18] This can make it difficult for parents to accept their baby may have an underlying health condition which can impact on treatment adherence and affect attendance at follow-up appointments. In addition, parental religious or cultural beliefs could also impact on parent's acceptance of their baby's suspected condition.[10 25] These results demonstrate the importance of always recording clear contact information for all relevant family members on the NBS card as well information about the language spoken, the need for a translator and any relevant religious or cultural information.

The impact on HPs of communicating of positive NBS results to families has rarely been considered. It has been acknowledged that the emotional management of families could lead to additional stress and anxiety.[26] However, HPs in the present study stated they found communicating positive NBS results to families difficult and emotive yet there were no formal mechanisms in place to support them. Despite this, HPs said they felt well supported by their colleagues. However, given the high levels of stress being reported by nurses and doctors and the reported rates of suicide amongst these professions[27 28] perhaps more consideration needs to be given to support staff undertaking such emotionally charged endeavours.

The general principles of communicating results that has emerged from this work could be extrapolated for other conditions where screening is recommended in children as well as breaking bad news in general. This might include conditions that may or may not be life altering/threatening but nevertheless can be distressing for parents. For example, delivering results of newborn hearing screening[29], findings from the physical examination of newborn babies (at birth and 6-8 weeks of age) including congenital cardiac abnormalities, congenital cataracts, cryptorchidism, developmental dislocation of the hip and findings from screening of children's eyes at 4-5 years of age. It may also be possible to extrapolate findings from the

For peer review only - http://bmjopen.bmj.com/site/about/guidelines.xhtml

present study for the delivery of bad news to parents in instances such as children newly diagnosed with cancer or following diagnosis of chronic conditions such as diabetes or epilepsy.

Strengths and Limitations

The current study has numerous strengths. HPs involved in the present study were employed in three different trusts; two in London and on in the West Midlands and were very experienced, supporting families for on average 10 years. Clinicians with less experience may have felt even more strongly about the need for clearer guidance. In addition, HPs were recruited from clinical teams involved in managing all of the conditions currently included in the NBS programme. This increases the transferability of the study findings as previous work has mainly focussed on CF and SCD. In addition, the study design, data collection and analysis were influenced by members of the PPI advisory group and relevant charities.

Participants were recruited via email; those with a pre-existing interest in this topic may have been more likely to self-select into the study. These people may communicate results differently than providers who did not participate in the study which may have based the findings.

The researchers are experienced in this field which may have biased data collection and analysis. Following the interviews, a HP event was held and the initial findings of the interviews were presented to the HPs who had been interviewed in each site. The purpose of this was to ensure that the analysis accurately represented their views. Participants agreed that the analysis was correct and priorities identified were accurate.

Recommendations for practice

The findings from this study suggest a number of recommendations for practice. For example, development of a competency framework for individuals involved in the process of communicating positive NBS results to families would ensure only HPs who are appropriately prepared undertake this task.

In addition, development of a standard laboratory form for communicating positive NBS results to clinical teams would ensure that when results are received from several laboratories by one clinical team, the information provided by each is consistent.

For peer review only - http://bmjopen.bmj.com/site/about/guidelines.xhtml

The development of condition specific checklists for HPs involved in communicating positive NBS result to families would ensure that vital information is consistently relayed to families and less experienced staff would be supported in terms of the information they need to provide. These could also act as an aide memoir for HPs as it is know that this can be a very distressing time for parents and so it would help them to remain focussed. In addition, this would ensure that clear contact information for all relevant family members including information about language spoken, translation needs and religious or cultural requirements could also be recorded and would be easily accessible for all member of the child and family's care team.

Guidance regarding reliable sources of further information for parents would also reduce alarm that can be caused by accessing unhelpful content on the internet immediately after the initial communication of the positive NBS result. This might include the use of specifically designed Apps or other forms of "easy to access" and helpful on-line information for parents.

Finally, regular clinical supervision and emotional support for all staff engaged in such work, should be encouraged to ensure staff are adequately supported to undertake this challenging task.

CONCLUSION

HPs invest a lot of time and energy trying to make sure communication of positive NBS results to families is done well. However, there continues to be great variation in the way positive NBS results are communicated to parents and this is largely influenced by resources available but also the lack of concrete guidance. Evidence based guidance derived from HPs' and parents' perspectives is needed to ensure there is parity in terms of communication practices. These should include, what training should be undertaken prior to undertaking this challenging role and guidelines related to what information should be relayed by whom and when. In addition, adequate support mechanism for HPs should be in place.

For peer review only - http://bmjopen.bmj.com/site/about/guidelines.xhtml

Acknowledgements: We would like to thank the Newborn Screening Laboratory Directors in England for agreeing to act as local principle investigators for this study as well as members of clinical teams who gave their valuable time. We would also like to thank all the parents in the Public and Patient Involvement Advisory Group for this study for their invaluable input.

Author Contributions:
JC made substantial contributions to the conception and design of the work. She acquired and interpreted the data for the work. She was involved in drafting the work, approved the final version to be published and agrees to be accountable for all aspects of the work in ensuring that questions related to the accuracy or integrity of any part of the work are appropriately investigated and resolved. HC acquired and interpreted the data for the work. She was involved in drafting the work, approved the final version to be published and agrees to be accountable for all aspects of the work in ensuring that questions related to the accuracy or integrity of any part of the work are appropriately investigated and resolved. JRB, AS and SM made substantial contributions to the conception and design of the work. They were involved in drafting the work, approved the final version to be published and agree to be accountable for all aspects of the work in ensuring that questions related to the accuracy or integrity of any part of the work are appropriately investigated and resolved. EKO was involved interpreting the data for the work and revising the work critically for important intellectual content, approved the final version to be published and agrees to be accountable for all aspects of the work in ensuring that questions related to the accuracy or integrity of any part of the work are appropriately investigated and resolved. LM, FU, MD and KWS made substantial contributions to the conception and design of the work. They were involved in drafting the work, approved the final version to be published and agree to be accountable for all aspects of the work in ensuring that questions related to the accuracy or integrity of any part of the work are appropriately investigated and resolved.

Funding: This study was supported by the National Institute for Health Research (NIHR) [Health Services and Delivery Research (project reference 16/52/25)]. The views expressed are those of the authors and not necessarily those of the NIHR or the Department of Health and Social Care.

For peer review only - http://bmjopen.bmj.com/site/about/guidelines.xhtml

Competing interests: None declared.

Ethics approval: This study was approved by the London Stanmore ethics committee, reference 17/LO/2102

Data sharing statement: Data are available upon reasonable request from the corresponding author subject to restrictions to preserve anonymity and personal privacy [JC]. These data are not publicly available as they contain information that could compromise research participant privacy/consent. Data will be available beginning 1 year and ending 5 years after publication to researchers who propose a methodologically sound proposal. Proposals should be directed to j.chudleigh@city.ac.uk. To gain access, data requesters will need to sign a data access agreement.

For peer review only

For peer review only - http://bmjopen.bmj.com/site/about/guidelines.xhtml

REFERENCES

1. UK Newborn Screening Programme Centre. Health Professional Handbook: A guide to newborn blood spot screening for healthcare professionals. London: UK Newborn Screening Programme Centre, 2012:1-56.
2. Bush A. Newborn screening for cystic fibrosis – benefit or bane? *Paediatric respiratory reviews* 2008;9:301-2.
3. Public Health England. Newborn Blood Spot Screening Programme in the UK: Data Collection and Performance Analysis Report 2016 to 2017. London, 2018:1-65.
4. Public Health England. NHS Sickle Cell and Thalassaemia Screening Programme Data Report 2015/16: Trends and performance analysis. London, 2017:1-81.
5. Public Health England. Newborn blood spot screening: programme handbook: Public Health England, 2018.
6. Salm A, Yetter E, Tluczek A. Informing parents about positive newborn screening results: Parents' recommendations *Journal of Child Health Care* 2012;16(4):367-81.
7. Ulph F, Cullinan T, Qureshi N, et al. Parents' responses to receiving sickle cell or cystic fibrosis carrier results for their child following newborn screening. *Eur J Hum Genet* 2015;23(4):459-65. doi: DOI 10.1038/ejhg.2014.126
8. Ulph F, Cullinan T, Qureshi N, et al. The impact on parents of receiving a carrier result for sickle cell or cystic fibrosis for their child via newborn screening. *Eur J Hum Genet* 2014;22
9. Tluczek A, Clark R, McKechnie AC, et al. Factors affecting parent-child relationships one year after positive newborn screening for cystic fibrosis or congenital hypothyroidism. *J Dev Behav Pediatr* 2015;36(1):24-34. doi: 10.1097/DBP.0000000000000112
10. Chudleigh J, Buckingham S, Dignan J, et al. Parents' Experiences of Receiving the Initial Positive Newborn Screening (NBS) Result for Cystic Fibrosis and Sickle Cell Disease. *J Genet Couns* 2016 doi: 10.1007/s10897-016-9959-4
11. Parker H, Qureshi N, Ulph F, et al. Imparting carrier status results detected by universal newborn screening for sickle cell and cystic fibrosis in England: a qualitative study of current practice and policy challenges. *BMC Health Serv Res* 2007;7:203. doi: 1472-6963-7-203 [pii] 10.1186/1472-6963-7-203 [published Online First: 2007/12/15]
12. Ulph F, Cullinan T, Qureshi N, et al. Parents' responses to receiving sickle cell or cystic fibrosis carrier results for their child following newborn screening. *Eur J Hum Genet* 2015;23(4):459-65. doi: 10.1038/ejhg.2014.126
13. Chudleigh J, Ren CL, Barben J, et al. International approaches for delivery of positive newborn bloodspot screening results for CF. *Journal of cystic fibrosis : official journal of the European Cystic Fibrosis Society* 2019;18(5):614-21. doi: 10.1016/j.jcf.2019.04.004
14. Kai J, Ulph F, Cullinan T, et al. Communication of carrier status information following universal newborn screening for sickle cell disorders and cystic fibrosis: qualitative study of experience and practice. *Health Technol Assess* 2009;13(57):1-82, iii. doi: 10.3310/hta13570 [published Online First: 2009/12/02]
15. Tluczek A, Koscik RL, Farrell PM, et al. Psychosocial risk associated with newborn screening for cystic fibrosis: parents' experience while awaiting the sweat-test appointment. *Pediatrics* 2005;115(6):1692-703. doi: 10.1542/peds.2004-0275
16. Collins JL, La Pean A, O'Tool F, et al. Factors that influence parents' experiences with results disclosure after newborn screening identifies genetic carrier status for cystic fibrosis or sickle cell hemoglobinopathy. *Patient Educ Couns* 2012 doi: S0738-3991(11)00606-9 [pii] 10.1016/j.pec.2011.12.007 [published Online First: 2012/01/14]
17. Buchbinder M, Timmermans S. Newborn screening for metabolic disorders: parental perceptions of the initial communication of results. *Clin Pediatr (Phila)* 2012;51(8):739-44. doi: 0009922812446011 [pii] 10.1177/0009922812446011 [published Online First: 2012/05/09]

For peer review only

18. DeLuca JM, Kearney MH, Norton SA, et al. Parents' experiences of expanded newborn screening evaluations. *Pediatrics* 2011;128(1):53-61. doi: peds.2010-3413 [pii] 10.1542/peds.2010-3413 [published Online First: 2011/06/29]
19. Rueegg CS, Barben J, Hafen GM, et al. Newborn screening for cystic fibrosis - The parent perspective. *Journal of cystic fibrosis : official journal of the European Cystic Fibrosis Society* 2016;15(4):443-51. doi: 10.1016/j.jcf.2015.12.003
20. Chudleigh JC, H. Bonham, J.R. Olander, E.K. Moody, L. Simpson, A. Morris, S. Ulph, F. Bryon, M. Southern, K.W. . A qualitative exploration of parents' experiences of receiving positive newborn bloodspot screening results for nine conditions in England. *In Press* 2020
21. Chudleigh J, Bonham J, Bryon M, et al. Rethinking Strategies for Positive Newborn Screening Result (NBS+) Delivery (ReSPoND): a process evaluation of co-designing interventions to minimise impact on parental emotional well-being and stress. *Pilot Feasibility Stud* 2019;5:108. doi: 10.1186/s40814-019-0487-5
22. Braun V, Clarke V. Using thematic analysis in psychology. *Qualitative Research in Psychology* 2006;3(2):77-101.
23. Milford C, Kriel Y, Njau I, et al. Teamwork in Qualitative Research: Descriptions of a Multicountry Team Approach. *International Journal of Qualitative Methods* 2017;16:1-10.
24. DeCuir-Gunby JT, Marshall PL, McCulloch AW. Developing and Using a Codebook for the Analysis of Interview Data: An Example from a Professional Development Research Project. *Field Methods* 2011;23(2):136-55.
25. Marsh VM, Kamuya DM, Molyneux SS. 'All her children are born that way': gendered experiences of stigma in families affected by sickle cell disorder in rural Kenya. *Ethn Health* 2011;16(4-5):343-59. doi: 10.1080/13557858.2010.541903
26. Moody L, Atkinson L, Kehal I, et al. Healthcare professionals' and parents' experiences of the confirmatory testing period: a qualitative study of the UK expanded newborn screening pilot. *BMC Pediatr* 2017;17(1):121. doi: 10.1186/s12887-017-0873-1
27. Gerada C. Doctors and suicide. *Br J Gen Pract* 2018;68(669):168-69. doi: 10.3399/bjgp18X695345
28. Selby A. More than 300 overworked NHS nurses have died by suicide in just seven years. *Daily Mirror* 2019.
29. Gilbey P. Qualitative analysis of parents' experience with receiving the news of the detection of their child's hearing loss. *Int J Pediatr Otorhinolaryngol* 2010;74(3):265-70. doi: 10.1016/j.ijporl.2009.11.017

For peer review only

For peer review only - http://bmjopen.bmj.com/site/about/guidelines.xhtml

Consolidated criteria for reporting qualitative studies (COREQ): 32-item checklist

Developed from:
Tong A, Sainsbury P, Craig J. Consolidated criteria for reporting qualitative research (COREQ): a 32-item checklist for interviews and focus groups. *International Journal for Quality in Health Care*. 2007. Volume 19, Number 6: pp. 349 – 357

YOU MUST PROVIDE A RESPONSE FOR ALL ITEMS. ENTER N/A IF NOT APPLICABLE

No. Item	Guide questions/description	Reported on Page #
**Domain 1: Research team and reflexivity**		
Personal Characteristics		
1. Inter viewer/facilitator	Which author/s conducted the inter view or focus group?	Section 2.5
2. Credentials	What were the researcher's credentials? E.g. PhD, MD	PhD
3. Occupation	What was their occupation at the time of the study?	Children's Nurse (JC) and Psychologist (HC)
4. Gender	Was the researcher male or female?	Female
5. Experience and training	What experience or training did the researcher have?	MSc Research Methods; extensive clinical experience; interview techniques.
Relationship with participants		
6. Relationship established	Was a relationship established prior to study commencement?	No
7. Participant knowledge of the interviewer	What did the participants know about the researcher? e.g. personal goals, reasons for doing the research	Professional role – Children's nurse, researcher role in this study
8. Interviewer characteristics	What characteristics were reported about the inter viewer/facilitator? e.g. Bias, assumptions, reasons and interests in the research topic	Section 5.0
**Domain 2: study design**		
Theoretical framework		
9. Methodological orientation and Theory	What methodological orientation was stated to underpin the study? e.g. grounded theory, discourse analysis, ethnography, phenomenology, content analysis	Qualitative exploratory design
Participant selection		

For peer review only

10. Sampling	How were participants selected? e.g. purposive, convenience, consecutive, snowball	Purposive
11. Method of approach	How were participants approached? e.g. face-to-face, telephone, mail, email	Email
12. Sample size	How many participants were in the study?	Seventeen
13. Non-participation	How many people refused to participate or dropped out? Reasons?	Three – time constraints / workload demands
Setting		
14. Setting of data collection	Where was the data collected? e.g. home, clinic, workplace	Workplace
15. Presence of non-participants	Was anyone else present besides the participants and researchers?	No
16. Description of sample	What are the important characteristics of the sample? e.g. demographic data, date	Staff involved in communicating the initial positive NBS result in the preceding 6 months.
Data collection		
17. Interview guide	Were questions, prompts, guides provided by the authors? Was it pilot tested?	Yes
18. Repeat interviews	Were repeat inter views carried out? If yes, how many?	N/A
19. Audio/visual recording	Did the research use audio or visual recording to collect the data?	Audio recording
20. Field notes	Were field notes made during and/or after the inter view or focus group?	Yes
21. Duration	What was the duration of the inter views or focus group?	37 minutes (range 19-58 minutes)
22. Data saturation	Was data saturation discussed?	Yes
23. Transcripts returned	Were transcripts returned to participants for comment and/or correction?	No but a staff meeting was held after all interviews had been conducted as per the protocol
**Domain 3: analysis and findings**		
Data analysis		
24. Number of data coders	How many data coders coded the data?	Two
25. Description of the coding tree	Did authors provide a description of the coding tree?	Yes
26. Derivation of themes	Were themes identified in advance or derived from the data?	Derived from the data
27. Software	What software, if applicable, was used to manage the data?	N/A
28. Participant checking	Did participants provide feedback on the findings?	Yes

For peer review only

Reporting		
29. Quotations presented	Were participant quotations presented to illustrate the themes/findings? Was each quotation identified? e.g. participant number	Results section
30. Data and findings consistent	Was there consistency between the data presented and the findings?	Yes
31. Clarity of major themes	Were major themes clearly presented in the findings?	Results
32. Clarity of minor themes	Is there a description of diverse cases or discussion of minor themes?	Discussion

Once you have completed this checklist, please save a copy and upload it as part of your submission. When requested to do so as part of the upload process, please select the file type: *Checklist*. You will NOT be able to proceed with submission unless the checklist has been uploaded. Please DO NOT include this checklist as part of the main manuscript document. It must be uploaded as a separate file.

For peer review only

BMJ Open

A qualitative exploration of health professionals' experiences of communicating positive newborn bloodspot screening results for nine conditions in England

Journal:	*BMJ Open*
Manuscript ID	bmjopen-2020-037081.R2
Article Type:	Original research
Date Submitted by the Author:	29-Jul-2020
Complete List of Authors:	Chudleigh, Jane; City University, School of Health Sciences<br>Chinnery, Holly; St Mary's University Twickenham<br>Bonham, Jim; Sheffield Children's NHS Foundation Trust<br>Olander, Ellinor; City University London<br>Moody, Louise; Coventry University<br>Simpson, Alan; King's College London,<br>Morris, Stephen; University of Cambridge School of Clinical Medicine, Primary Care Unit<br>Ulph, Fiona; University of Manchester<br>Bryon, Mandy; Great Ormond Street Hospital For Children NHS Foundation Trust<br>Southern, Kevin; Institute of Child Health, Paediatrics
**Primary Subject Heading**:	Communication
Secondary Subject Heading:	Paediatrics
Keywords:	GENETICS, PAEDIATRICS, Cystic fibrosis < THORACIC MEDICINE, HAEMATOLOGY, Other metabolic, e.g. iron, porphyria < DIABETES & ENDOCRINOLOGY

SCHOLARONE™
Manuscripts

I, the Submitting Author has the right to grant and does grant on behalf of all authors of the Work (as defined in the below author licence), an exclusive licence and/or a non-exclusive licence for contributions from authors who are: i) UK Crown employees; ii) where BMJ has agreed a CC-BY licence shall apply, and/or iii) in accordance with the terms applicable for US Federal Government officers or employees acting as part of their official duties; on a worldwide, perpetual, irrevocable, royalty-free basis to BMJ Publishing Group Ltd ("BMJ") its licensees and where the relevant Journal is co-owned by BMJ to the co-owners of the Journal, to publish the Work in this journal and any other BMJ products and to exploit all rights, as set out in our licence.

The Submitting Author accepts and understands that any supply made under these terms is made by BMJ to the Submitting Author unless you are acting as an employee on behalf of your employer or a postgraduate student of an affiliated institution which is paying any applicable article publishing charge ("APC") for Open Access articles. Where the Submitting Author wishes to make the Work available on an Open Access basis (and intends to pay the relevant APC), the terms of reuse of such Open Access shall be governed by a Creative Commons licence – details of these licences and which Creative Commons licence will apply to this Work are set out in our licence referred to above.

Other than as permitted in any relevant BMJ Author's Self Archiving Policies, I confirm this Work has not been accepted for publication elsewhere, is not being considered for publication elsewhere and does not duplicate material already published. I confirm all authors consent to publication of this Work and authorise the granting of this licence.

For peer review only

TITLE PAGE

A qualitative exploration of health professionals' experiences of communicating positive newborn bloodspot screening results for nine conditions in England

Jane Chudleigh[1]*, Holly Chinnery[2], Jim R Bonham[3], Ellinor K Olander[1], Louise Moody[4], Alan Simpson[5], Stephen Morris[6], Fiona Ulph[7], Mandy Bryon[8], Kevin W Southern[9]

*corresponding author

[1]Centre for Maternal and Child Health Research, City, University of London, EC1V 0HB, UK;

[2]Faculty of Sports, Health and Applied Science, St Mary's University Twickenham, TW1 4SX

[3]Division of Pharmacy, Diagnostics and Genetics Sheffield Children's NHS Foundation Trust, S10 2TH, UK;

4 Centre for Arts, Memory and Communities, Coventry University, CV1 5FB, UK;

[5] Florence Nightingale Faculty of Nursing, Midwifery & Palliative Care, King's College London SE1 8WA, UK; [6] University of Cambridge School of Clinical Medicine, CB2 0SP, UK; [7]Division of Psychology and Mental Health, The University of Manchester, M13 9PL, UK, [8]Paediatric Psychology and Play Services, Great Ormond Street Hospital for Children NHS Foundation Trust, WC1N 3JH, UK, [9] Women's and Children's Health, University of Liverpool, L69 3BX, UK

j.chudleigh@city.ac.uk; holly.chinnery@stmarys.ac.uk; jim.bonham@sch.nhs.uk; Ellinor.Olander@city.ac.uk; l.moody@coventry.ac.uk; alan.simpson@kcl.ac.uk; sm2428@medschl.cam.ac.uk; fiona.ulph@manchester.ac.uk; mandy.bryon@gosh.nhs.uk; kwsouth@liv.ac.uk

For peer review only

For peer review only - http://bmjopen.bmj.com/site/about/guidelines.xhtml

ABSTRACT

Objective: To explore health professionals experiences of communicating positive NBS results, highlight differences, share good practice and make recommendations for future research.

Design: Qualitative exploratory design was employed using semi-structured interviews

Setting: Three National Health Service provider organisations in England

Participants: Seventeen health professionals involved in communicating positive newborn bloodspot screening results to parents for all nine conditions currently included in the newborn bloodspot screening programme in England.

Results: Findings indicated variation in approaches to communicating positive newborn bloodspot screening results to parents, largely influenced by resources available and the lack of clear guidance. Health professionals emphasised the importance of communicating results to families in a way that is sensitive to their needs. However, many challenges hindered communication including logistical considerations; difficulty contacting the family and other health professionals; language barriers; parental reactions; resource considerations; lack of training; and insufficient time.

Conclusion: Health professionals invest a lot of time and energy trying to ensure communication of positive newborn bloodspot screening results to families is done well. However, there continues to be great variation in the way these results are communicated to parents and this is largely influenced by resources available but also the lack of concrete guidance. How best to support health professionals undertaking this challenging and emotive task requires further exploration. We recommend evaluation of a more cohesive approach that meets the needs of parents and staff while being sensitive to the subtleties of each condition.

Trial registration: ISRCTN 15330120

Key words: Newborn bloodspot screening, communication

Word count: 5935

For peer review only - http://bmjopen.bmj.com/site/about/guidelines.xhtml

Strengths and limitations of this study

- Health professionals involved in the present study were employed in three different NHS organisations, increasing transferability of the findings, and were very experienced, supporting families for on average 10 years
- Participants were recruited via email; those with a pre-existing interest in this topic may have been more likely to self-select into the study. These people may communicate results differently than providers who did not participate in the study
- Health professionals were recruited from clinical teams involved in managing all nine conditions currently included in the NBS programme in England; previous work has mainly focussed on cystic fibrosis and sickle cell disease
- The study design, data collection and analysis were influenced by members of the patient and public involvement advisory group and relevant charities

For peer review only

INTRODUCTION

Newborn bloodspot screening (NBS) seeks to identify pre-symptomatic babies that are affected by genetic or congenital conditions[1]; early diagnosis leads to better health outcomes for the child[1 2]. In January 2015, NBS in England and Wales expanded and now covers nine conditions: sickle cell disease (SCD); cystic fibrosis (CF); phenylketonuria (PKU); homocystinuria (HCU); glutaric aciduria type 1 (GA1) medium chain acyl CoA dehydrogenase deficiency (MCADD), maple syrup urine disease (MSUD), isovaleric aciduria (IVA) and Congenital Hypothyroidism (CHT).

In the UK, parents are given information prior to and after birth and are asked to provide informed consent for their baby to take part in the NBS Programme. NBS involves a small amount of blood being taken from the baby's heel on day 5 of life. Each year, around 10,000 parents[1] of babies born in the UK are given a positive NBS result. This happens around 2-8 weeks after birth, depending on the condition[3 4]. Parents are informed of the NBS result, normally on the same day it becomes available, and are asked to bring their baby to a particular hospital for diagnostic testing within a given timeframe depending on the suspected condition.

National Guidance regarding the content and mode of communication between health professionals (HPs) and parents is generic and vague[1 5]. Communication of positive NBS results is often delivered by specialist nurses and is a subtle and skilful task which demands thought, preparation and evidence to minimise potentially harmful negative sequela[6-10]. However, training for this role is challenging due to the fact that for many of conditions, a positive result occurs relatively infrequently and may be communicated by several members of the appropriate team of people.

There is evidence of variations both nationally and internationally with regard to the approaches used to communicate positive NBS results particularly in relation to CF and SCD[10-13]. These variations include the different approaches used to communicate the results (such as in person during a home visit or by letter)[11 14], the choice of person or profession to deliver the result and the content of the communication[15-19].

[1] The term 'parents' refers to parents, carers or guardians.

For peer review only

The findings of Kai et al's study[14] informed the development of the current national guidelines for the communication process in the NBS programme[1 5], which recommend face-to-face communication by an appropriately trained HP. Despite these guidelines, a study reporting the findings from 67 interviews with parents about their experience of receiving CF or SCD carrier results following NBS indicated that disparity continues to exist regarding how the guidelines are implemented in practice[7]. The findings also indicated variability in the content and the way the result was communicated which led to increased parental anxiety and distress; the perceived lack of knowledge of the person communicating the result was potentially more distressing than the positive finding [7]. This highlights the importance of understanding varied practice in order to identify, share and recommend good practice.

The purpose of this work was to explore HPs' experiences of communicating positive NBS results to highlight differences, share good practice and make recommendations for future research. Experiences of parents are reported elsewhere[20].

METHODS

A qualitative exploratory design was employed using semi-structured interviews as part of a large ongoing programme of work[21] . The study was approved by the London Stanmore ethics committee (17/LO/2102).

Setting

Study sites consisted of three National Health Service provider organisations in England that process comparable numbers of positive NBS reports annually for each of the nine conditions currently included in the NBS Programme. These consisted of two in Greater London (served by one NBS laboratory processing 128 positive NBS results in 2017/18) and one in the West Midlands (whose NBS laboratory processed 129 positive NBS results in 2017/18).

Patient and public involvement

Patient and public involvement (PPI) was instrumental in the design and conduct of this work. Parents of babies who had received a positive NBS screening result for one of the nine screened conditions formed a PPI advisory group who met prior to, during and following data collection. Their suggestions were incorporated into the study design, the data collection tools and the data analysis and presentation. In addition, views of representatives from charities for the screened conditions including Metabolic Support UK, the British Thyroid Foundation, the

For peer review only - http://bmjopen.bmj.com/site/about/guidelines.xhtml

CF Trust and the Sickle Cell Society were also sought. These groups were able to provide advice on sampling methods, choice of study sites and the analysis and presentation of parental interview responses.

Inclusion and Exclusion Criteria

HPs involved in communicating positive NBS results in the previous 6 months were invited to take part in the study. HPs who had not been involved in communicating positive NBS results in the last 6 months or who had personal experience of receiving a positive NBS result were excluded.

Recruitment and Sampling

A two-stage sampling approach was employed where participants were first sampled purposively based on their experience with the phenomena of interest, followed by a second stage of snowball sampling where the first participants suggested others. Members of relevant clinical teams (medical consultants; general paediatricians; nurse specialists; specialist screening nurses) were initially identified through individual Trust websites and contacted via email and invited to participate. If no response was received, a follow up email was sent after one week. Identified HPs were asked if there were any other members of the clinical teams that the research team should contact to ensure views were representative. All potential participants were given the choice to participate or not and were reminded of their right to withdraw from the study at any time. Written informed consent was obtained from all participants ahead of the interview.

Data Collection

Semi-structured, face-to-face interviews comprising closed and open-ended questions were conducted by JC and HC from September 2018 to March 2019, to explore HPs experiences of communicating positive NBS to families. Interviews took place at the participants' place of work in a location and at a time of their choice. Topics covered included how HPs manage communication, examples of situations when it has gone well or not as well as expected and the possible reasons for this. We also explored how participants felt during the experience and asked for any suggested changes to practice. Interviews were audio recorded and transcribed verbatim.

For peer review only - http://bmjopen.bmj.com/site/about/guidelines.xhtml

For peer review only

Data Analysis

Interviews were analysed for themes[22]. An inductive approach to data analysis was used and themes generated using a latent approach[22] to provide a deeper understanding of approaches used to communicate positive NBS results to families. The six phases of thematic analysis described by Braun and Clarke[22] guided data analysis.

Two members of the research team (JC and HC) coded one interview transcript separately. These codes were then compared to inform and align code development[23] and a code book was developed. A further four transcripts were then coded separately by the same two members of the research team using the code book. These separately coded transcripts were then compared; inter-coder reliability was 84%. Following this, the same two members of the research team coded the remainder of the transcripts using the code book. Once this initial coding had been completed, all data for each code were compared to ensure consistency in coding and to enable the codes to be collapsed into themes. All quotes for each theme were collated to inform theme development. This was an ongoing, iterative process; new codes were developed and the definition of codes refined as analysis progressed.[24]

Positionality and reflexivity

Members of the study team (JC, JRB, LM, FU, MB, KWS) have been involved in or continue to undertake a variety of roles and activities associated with the NBS Programme in the UK. It is acknowledged that this could have led to potential bias during data collection and analysis. However, this was balanced by other members of the research team who had previously had minimal involvement in NBS (HC, EKO, AS, SM). Data collection and analysis was mainly undertaken by JC and HC who fall within both camps. Neither JC nor HC were employed in the same organisations where data collection was undertaken.

RESULTS

In total, 20 HPs were emailed and invited to participate. Two HPs did not respond to the invitation and one HP did not communicate the initial positive screening result and was therefore ineligible. Therefore, 16 face to face interviews were conducted with 17 HPs (two requested to be interviewed together); eight were from the West Midlands and the remaining nine were split across the two Greater London Trusts. Participants with experience of all the nine screened conditions were included. Interviews lasted on average 37 minutes (range 19-

For peer review only - http://bmjopen.bmj.com/site/about/guidelines.xhtml

58 minutes). The sample consisted of 8 medical consultants, 1 medical registrar, 7 nurse specialists / advanced nurse practitioners and 1 screening nurse. Length of experience with newborn screening ranged from 2-38 years (median 8 years)

Five themes were identified: communication between HPs; process of communicating with the family; parent and family centred care; availability of resources and challenges to effective communication. Illustrative quotations are used to support the themes.

Communication between HPs

HPs reported a range of communication approaches to ensure sufficient information was available to them prior to communicating with families. This started with the laboratory communicating the result to the relevant clinical team in a variety of ways. These included a letter, normally via email or a telephone call followed by an email or a personal visit from a member of the screening laboratory to the clinical nurse specialist, the screening nurse, the on-call consultant or the named consultant depending on the condition, local resources and agreements.

"So we tend to find out from the newborn screening nurses. So, they'll be alerted by the labs and then they would give us [the physician] a call"[SS1P202]

The written initial communication consisted of a pro forma which was often developed locally and may or may not have been accompanied by a copy of the NBS card.

"...generally, we get a pro forma from the screening lab that's slightly different I think depending on the screening lab."[SS2P206]

"We actually started asking for the card."[SS2P201&02]

Receiving a copy of the NBS card was viewed favourably as it enabled HPs to check referral information and parental contact details if this were found to be ambiguous in any way.

Often, this would initiate a two pronged approach where HPs would commence gathering additional information about the child and family from health visitors (registered nurses or midwives who have undertaken additional training and work mainly with children from birth

For peer review only - http://bmjopen.bmj.com/site/about/guidelines.xhtml

to 5 years and their families), midwives and/or primary care physicians before contacting the family.

"Sometimes, it is good to know, the family dynamics, social care issues etc. from somebody [the health visitor or midwife] who's already involved with the family". [SS1P203]

However, clinicians sometimes found it challenging to make contact with health visitors and/or midwives to gather additional information about the family.

"…quite often we are leaving messages to ask them [the health visitor or midwife] to call us back and quite often we are receiving those calls back, after we've already visited the family." [SS1P203]

Simultaneously, members of the multi professional team would also be contacted such as the physiotherapist, dietician or pharmacist (depending on the condition) to inform them that the child would be attending the hospital either the same or the following day.

"The CF nurse specialist would let other members of the team know, so the physio and dietician know that there was a new baby, positive screen so that they could be on standby, but not necessarily to see the family."[SS1P201]

Similarly, if the plan was to see the child in a local hospital rather than in a tertiary care centre, similar communication would happen between the specialist centre and local clinical teams.

Process of communicating with the family

The initial contact with the family was undertaken in a variety of ways by different members of the clinical team. This included, face to face contact in the family's home, via telephone, text message or letter from the screening nurse, relevant clinical nurse specialist or medical consultant. Some respondents commented that they felt the person who told the family should not be a member of the specialist clinical team who would go on to care for the child and they felt this may taint any ongoing relationship. Others felt it was important that the person who gave the initial positive NBS result should be part of the child's clinical team to start building

For peer review only - http://bmjopen.bmj.com/site/about/guidelines.xhtml

familiarity and continuity of care or that there should be someone present who is known to the family.

"So the ideal is that either the midwife or the health visitor can come on the visit so that somebody in the family already knows." [SS1P203]

Views regarding how the initial communication with the family was conducted varied. Some clinicians felt it should be face-to face as it allowed the clinician to gather information about the family that would help to inform the follow-up visit at the hospital with the clinical team.

"You just don't know what's going on in the home environment and you've, sort of, been and witnessed it for yourself and it just gives you a good insight into the family dynamics or what's going on or what support mechanism are in place. You can't get that over the telephone." [SS1P202].

Others felt that a telephone call would be more appropriate to ensure families were told as soon as possible while some felt that a text message asking the family to call them back had worked really well and that a home visit may be quite intrusive. This suggests that sometimes, the approach to communication may not be steered wholly by the needs of the family but sometimes the experience of the person communicating the result.

"You can't really get rid of anyone in someone's house, can you? …You'd be a bit like, 'Right, go now from my house. Get out of my house'...how scary must it be for somebody to turn up on your doorstep"[SS2P201&02]

Regardless of who or how the initial communication took place, all respondents acknowledged the importance of 'getting it right'. Respondents felt it was important the person giving the positive screening result to the family was knowledgeable about the condition they would be discussing with the family. Indeed, many felt this was more important than how the information was delivered and could influence perceptions of ongoing care.

"I think the most important thing, in my opinion, is that the person giving that information, the first time, needs to be someone that can answer some questions." [SS1P207]

For peer review only - http://bmjopen.bmj.com/site/about/guidelines.xhtml

"I think that first telephone call and the first time you see them is absolutely critical…I think the family's views of what's going to come next will be completely modified by how it's done and their confidence in the service." [SS2P206]

However, it was also acknowledged that although various guidelines and protocols existed for the laboratory staff when processing the NBS card and for treatment and management once the child had been diagnosed, there was a paucity of guidance regarding communication of the initial positive NBS result.

"We've got all the protocols around timelines and KPIs etc. but the one bit we don't have anything concrete about is who breaks the news, what level of training or experience they need to have before they do it and what the expectation is of what they should cover in that visit." [SS1P203]

Consequently, the content of the initial communication also varied considerably. Some clinicians spoke about having a template they followed which helped them to ensure they imparted all the information required during what could sometimes be a very emotive interaction. Most agreed that they would try to keep the information about the suspected condition during the initial communication quite brief due to the fact it is a screening result and therefore it would not be appropriate to give too much information about a condition that had not been confirmed. In addition, families were often perceived as being unable to absorb the information very well due to the shock of an unexpected result. Finally, clinicians knew that when families are seen the same or following day, they would receive a lot more information and therefore were reluctant to overload them with information during the first contact.

"So we always have said, 'Screening will be rechecked. The bloods will be redone.'" [SS1P203]

"We don't usually give them much information, they're [the family] usually really upset." [SS2P201&02]

For peer review only - http://bmjopen.bmj.com/site/about/guidelines.xhtml

However, HPs did recognise the importance of signposting families to additional information sources such as charity websites and the National NBS website following the initial communication and gave examples of excellent practice.

"We always take out suspected leaflets. They always get a copy of something to read. We take out a map for the hospital…so they know where to go to in that building….We leave them a letter that confirms what the screening result is and what that might mean for the baby. On the top of that letter, it has our mobile number and office number to give them permission to ring us if they were at all worrying."[SS1P203]

Parent and family centred care

The importance of having a parent and family-centred approach when communicating the positive NBS result was emphasised by all clinicians and was considered to be an example of excellent practice. HPs spoke about the importance of the content of the initial communication. This was subdivided into a beginning, namely, remembering to congratulate parents on the birth of their child. This was followed by a section focussed on tailoring the quantity and level of information given depending on the parental response, not giving too much information or overwhelming parents, being honest and providing emotional support and reassurance where needed.

"So, you just have to judge how much information to give them and how best to support them because if parents are crying and upset about the diagnosis, you obviously have to support them in a different way to other parents." [SS1P205]

Finally, ending with a positive message, giving parents' time and making sure the family know that the baby's mum should be encouraged to bring a support person to the first visit. Also, the importance of treating each family as individuals and acknowledging the enormity of the task they are undertaking.

"I think it's easy, particularly if you're tired or something, to appear a bit more routine, run of the mill, whereas, obviously, it's a really big thing. This is their child, and this is an important piece of information, and it matters hugely to them. You have to try and reflect that

For peer review only - http://bmjopen.bmj.com/site/about/guidelines.xhtml

in how you talk to them, rather than them just being another parent of another sickle baby, which is the danger."[SS3P206]

Some clinicians said they endeavour to find out how much parents already know for instance if they have another child with the condition or if they have been searching the internet before embarking on any explanations. Also, gauging how much families want to know as well as how much families are actually absorbing and when it is time to stop.

"…there are some families that come in that want to know every single detail about everything and you try and be systematic in how you deliver that information. There are other parents that you can tell, although you're trying to tell them things, they are not really taking it on board." [SS1P205]

Finally, HPs recognised the importance of supporting the whole family and therefore not making it the responsibility of the parents to share information or educate other family members.

"We're there to support whoever. The home visit follow-up that I do, I say, 'All are welcome, you know, if granny and granddad want to be there, if they've got questions, I'm happy to speak with them.' Very often, grandparents, aunts and uncles are there, you know. I've been to one and people have been there on mass, but that's their family support network and if that's usually there in place, we don't exclude anyone. I'm happy to talk to any family member." [SS1P202]

Availability of resources

HPs at all levels and for all conditions acknowledged a lack of training and related competencies in terms of breaking bad news to families. This resulted in screening nurses in particular developing their own training programme to address this deficit.

"…we've had to develop some competencies….but because it's so unique and there aren't other nurses funded through a lab post, that there aren't competencies around." [SS1P203]

Resources available at different hospitals also influenced communication of the initial positive NBS result. Therefore, even if clinicians felt that offering to do a home visit to

For peer review only - http://bmjopen.bmj.com/site/about/guidelines.xhtml

deliver the initial positive NBS result would be beneficial, the need to prioritise resources often meant this would not be an option.

"…we don't have the capacity [to do home visits]….I think it will be good if we can do that, but we definitely don't have the capacity for that." [SS2P203]

One hospital had screening nurses and part of their role was to deliver the initial positive NBS result to families, usually face-to-face in their home. In the other two hospitals, this resource was not available and therefore the responsibility of delivering the initial positive NBS result to families stayed with the relevant clinical team. Even though HPs who were able to, felt very positive about the ability to offer home visits, these were resource intensive particularly when compared to the option to contact parents via telephone.

"Probably around 40 minutes is a quick visit. The longest visit I have been there [excluding travel] is probably about three hours."[SS1P203]

The ability to offer timely follow-up appointments with the clinical team both due to clinical need and also to alleviate parental anxiety was also viewed as important. However, many respondents discussed the availability of resources as a potential barrier to this and this seemed particularly evident for babies with SCD.

"It can be a week if we've got a slot, or it can be anything up to four to five weeks….I think it is a very stressful time and some families find that too long." [SS1P209]

For other conditions, clinical need meant that families were seen almost immediately by the clinical team after they had been given the initial positive NBS result.

"So, it's all clinically indicated….the rest of the metabolic conditions will be seen the same day." [SS1P203]

Although ensuring families are seen quickly after being told their child has a positive NBS result was seen as important by the clinical team, this also posed problems on occasions in terms of the financial burden for families needing to travel to the hospital at short notice. Currently, there is no budget to prospectively pay for families' travel expenses to attend

For peer review only - http://bmjopen.bmj.com/site/about/guidelines.xhtml

clinic appointments which means that families are expected to meet these costs in the first instance. Although it may be possible, in certain circumstances, to apply to have these reimbursed at a later date, this does not help families who struggle to pay these costs up front.

"…but we have had families who, it has happened where a family said, 'I just don't think I have the money. I can't afford to come' and saying, 'We'll pay you back' doesn't help and that is difficult" [SS2P206].

Some clinicians also felt that the setting within which the family met the clinical team was important in terms of first impressions.

"This room was designed that it hasn't got a computer or a phone, unlike our other consultation rooms. So, it's a quiet space to deliver the news to the family." [SS1P202]

Availability of resources for diagnostic testing was also viewed as potentially problematic.

"…when we started, the labs were kitted out, such that they would be able to offer a sweat test and a result the same day, any day….because of cuts…they're much less flexible in terms of what they can offer." [SS1P201]

Therefore, although HPs felt very strongly about offering a parent-centred approach to communicating positive NBS results to parents, availability of training, staff and physical space could act as a barrier.

Other concerns related to practical and resource considerations included adding new conditions to the NBS Programme and changes in geographical areas covered by screening laboratories particularly in relation to inherited metabolic diseases and CHT.

"…they [babies with CHT] are dealt with by fifteen different centres…and I think that causes the labs quite a lot of problems. The labs would much prefer fewer centre." [SS2P207]

Challenges to effective communication

For peer review only - http://bmjopen.bmj.com/site/about/guidelines.xhtml

HPs in the current study were asked about their experience of communicating positive NBS result to families. Several staff commented on the personal and emotional impact of this aspect of their role.

"Some can be pretty traumatic and you just feel like you've destroyed their world." [SS1P202]

"It's very emotional for me sometimes, very, very emotional…the mother, a new born, holding a new born baby crying…based on the result you gave them…you wish you could change the result for them." [SS3P211]

Despite this, no formal mechanisms were in place to support HPs. However, all reported that they had developed their own support mechanisms within their teams.

"…everyone's very used to doing it, so everyone completely gets it and completely understands….you have a bit of a debrief with the consultant who's seeing the family." [SS3P202]

HPs also struggled to make contact with parents for a variety of reasons including, the contact details on the NBS card being illegible, parents having moved or staying with relatives after the NBS sample was taken and parents not answering their phones.

" You fill in clearly….not flipping scribbled…the name of the person, a first number, a second number of a partner or someone like that…even though we had a picture of the card, they hadn't filled in any of the details" [SS2P201&02]

"It's not being able to get in contact with people, because they're not picking up on a random London number…The amount of junk calls we all get nowadays, these people are just not picking up the phone." [SS2P201&02]

Once parents had been contacted, other challenges arose related to parental attributes. A common theme related to communicating with parents who did not speak English as a first language. This was not always apparent until after the clinician had attempted to make contact with the parent. This led to parents not understanding what was being communicated,

For peer review only - http://bmjopen.bmj.com/site/about/guidelines.xhtml

For peer review only

the seriousness of what was being communicated or simply not engaging in the conversation due to lack of understanding.

"If English isn't the first language, I certainly find it much more difficult to reassure, and try to be empathetic because you're worried about just getting the basic understanding across." [SS2P206]

Managing families who do not believe the diagnosis either because their child has no signs or symptoms or due to religious or cultural beliefs was also difficult. Such beliefs may also affect engagement with recommended medication and/or treatments. This was particularly evident for those families with a child with SCD.

"They just wouldn't accept that they'd got sickle, because they looked well." [SS1210]

"…so you get a few that either don't believe the diagnosis or their cultural beliefs are that God will mean that they don't have to do anything medical that we suggest." [SS1P205]

Clinicians commented that this often led to patients not attending clinical appointments which also posed challenges in terms of monitoring.

Oher challenges related directly to the NBS Programme. The NBS Programme is designed to identify babies with a higher chance of being affected by one of the screened conditions, often before they are symptomatic, so they can start treatment as soon as possible if needed, and therefore improve outcomes. However, the need to inform parents that their baby has a positive NBS result for a potentially life changing and/or life limiting condition when the baby is asymptomatic was challenging.

"You come to see the doctor because your child is unwell, whereas, this is the opposite, in that, you've got a child who appears perfectly healthy, and you're telling them that they're unwell." [SS1P207]

DISCUSSION

For peer review only - http://bmjopen.bmj.com/site/about/guidelines.xhtml

This study furthers our understanding of HPs' experiences of communicating positive NBS results to families. It is clear that staff involved in communication of positive NBS results are passionate about making sure that although the message is distressing for parents, it is done well. Variation in communication practices continue to exist and is influenced by many factors including resources available but also the lack of clear guidance. This impacted on the methods used to communicate positive NBS results but also the content of the communication to parents. This is supported by previous research which has been conducted both nationally and internationally[7 10 11 13] and suggests that further guidance may be needed to ensure a more cohesive approach which meets the needs of parents' and staff while being sensitive to the subtleties of each condition. However, the issue of finite resources and the need to prioritise these also needs careful consideration.

An overarching message from the HPs involved in the present study was the desire to ensure communicating positive NBS result to families is parent and family-centred. Once HPs were aware of a positive NBS result, they spent a great deal of time 'setting the scene' by gathering information from various sources in preparation for speaking with the family and organising the follow-up appointment with the clinical team. Often this important and necessary work would be time consuming and labour intensive in terms of identifying individuals who either obtained the original sample (midwives) or might have additional information about family dynamics (health visitor) and may be hindered by poor completion or lack of information on the NBS card.

Current guidance[1 5] does not explicitly state who is the 'right' person to communicate a positive NBS result to families or what training or qualifications they should have and specific training to undertake this role is not available. While this does allow flexibility in terms of resources, it can lead to disparity in terms of parental experience of receiving the NBS result. There is no specific guidance regarding exactly what information should be shared with parents during the initial communication[5]. While some HPs alluded to using an informal checklist, this was not universal and therefore may not lead to consistency between clinicians. However, clinicians did recognise the importance of the person imparting the result, having adequate condition specific knowledge, this is consistent with previous research[15 16 19]. These are common problems that have been highlighted both nationally and internationally.[13] As these are not dependent on specific healthcare systems *per se*, the

For peer review only - http://bmjopen.bmj.com/site/about/guidelines.xhtml

For peer review only

findings of the present study could also be extrapolated to screening programmes in other countries.

Clinicians experienced many challenges which hindered the communication of positive NBS results to families. This often stemmed from inadequate information on the NBS card but also parental reactions which could hinder effective communication. When parents are told the NBS result, their baby is often pre-symptomatic as this is one of the purposes of the NBS Programme which means the result is often unexpected.[10 17 18] This can make it difficult for parents to accept their baby may have an underlying health condition which can impact on treatment adherence and affect attendance at follow-up appointments. In addition, parental religious or cultural beliefs could also impact on parent's acceptance of their baby's suspected condition.[10 25] These results demonstrate the importance of always recording clear contact information for all relevant family members on the NBS card as well information about the language spoken, the need for a translator and any relevant religious or cultural information.

The impact on HPs of communicating of positive NBS results to families has rarely been considered. It has been acknowledged that the emotional management of families could lead to additional stress and anxiety.[26] However, HPs in the present study stated they found communicating positive NBS results to families difficult and emotive yet there were no formal mechanisms in place to support them. Despite this, HPs said they felt well supported by their colleagues. However, given the high levels of stress being reported by nurses and doctors and the reported rates of suicide amongst these professions[27 28] perhaps more consideration needs to be given to support staff undertaking such emotionally charged endeavours.

The general principles of communicating results that has emerged from this work could be extrapolated for other conditions where screening is recommended in children as well as breaking bad news in general. This might include conditions that may or may not be life altering/threatening but nevertheless can be distressing for parents. For example, delivering results of newborn hearing screening[29], findings from the physical examination of newborn babies (at birth and 6-8 weeks of age) including congenital cardiac abnormalities, congenital cataracts, cryptorchidism, developmental dislocation of the hip and findings from screening of children's eyes at 4-5 years of age. It may also be possible to extrapolate findings from the

For peer review only - http://bmjopen.bmj.com/site/about/guidelines.xhtml

present study for the delivery of bad news to parents in instances such as children newly diagnosed with cancer or following diagnosis of chronic conditions such as diabetes or epilepsy.

Strengths and Limitations

The current study has numerous strengths. HPs involved in the present study were employed in three different trusts; two in London and on in the West Midlands and were very experienced, supporting families for on average 10 years. Clinicians with less experience may have felt even more strongly about the need for clearer guidance. In addition, HPs were recruited from clinical teams involved in managing all of the conditions currently included in the NBS programme. This increases the transferability of the study findings as previous work has mainly focussed on CF and SCD. In addition, the study design, data collection and analysis were influenced by members of the PPI advisory group and relevant charities.

Participants were recruited via email; those with a pre-existing interest in this topic may have been more likely to self-select into the study. These people may communicate results differently than providers who did not participate in the study which may have based the findings.

The researchers are experienced in this field which may have biased data collection and analysis. Following the interviews, a HP event was held and the initial findings of the interviews were presented to the HPs who had been interviewed in each site. The purpose of this was to ensure that the analysis accurately represented their views. Participants agreed that the analysis was correct and priorities identified were accurate.

Recommendations for practice

The findings from this study suggest a number of recommendations for practice. For example, development of a competency framework for individuals involved in the process of communicating positive NBS results to families would ensure only HPs who are appropriately prepared undertake this task.

In addition, development of a standard laboratory form for communicating positive NBS results to clinical teams would ensure that when results are received from several laboratories by one clinical team, the information provided by each is consistent.

For peer review only - http://bmjopen.bmj.com/site/about/guidelines.xhtml

The development of condition specific checklists for HPs involved in communicating positive NBS result to families would ensure that vital information is consistently relayed to families and less experienced staff would be supported in terms of the information they need to provide. These could also act as an aide memoir for HPs as it is know that this can be a very distressing time for parents and so it would help them to remain focussed. In addition, this would ensure that clear contact information for all relevant family members including information about language spoken, translation needs and religious or cultural requirements could also be recorded and would be easily accessible for all member of the child and family's care team.

Guidance regarding reliable sources of further information for parents would also reduce alarm that can be caused by accessing unhelpful content on the internet immediately after the initial communication of the positive NBS result. This might include the use of specifically designed Apps or other forms of "easy to access" and helpful on-line information for parents.

Finally, regular clinical supervision and emotional support for all staff engaged in such work, should be encouraged to ensure staff are adequately supported to undertake this challenging task.

CONCLUSION

HPs invest a lot of time and energy trying to make sure communication of positive NBS results to families is done well. However, there continues to be great variation in the way positive NBS results are communicated to parents and this is largely influenced by resources available but also the lack of concrete guidance. Evidence based guidance derived from HPs' and parents' perspectives is needed to ensure there is parity in terms of communication practices. These should include, what training should be undertaken prior to undertaking this challenging role and guidelines related to what information should be relayed by whom and when. In addition, adequate support mechanism for HPs should be in place.

For peer review only - http://bmjopen.bmj.com/site/about/guidelines.xhtml

Acknowledgements: We would like to thank the Newborn Screening Laboratory Directors in England for agreeing to act as local principle investigators for this study as well as members of clinical teams who gave their valuable time. We would also like to thank all the parents in the Public and Patient Involvement Advisory Group for this study for their invaluable input.

Author Contributions:

JC made substantial contributions to the conception and design of the work. She acquired and interpreted the data for the work. She was involved in drafting the work, approved the final version to be published and agrees to be accountable for all aspects of the work in ensuring that questions related to the accuracy or integrity of any part of the work are appropriately investigated and resolved. HC acquired and interpreted the data for the work. She was involved in drafting the work, approved the final version to be published and agrees to be accountable for all aspects of the work in ensuring that questions related to the accuracy or integrity of any part of the work are appropriately investigated and resolved. JRB, AS and SM made substantial contributions to the conception and design of the work. They were involved in drafting the work, approved the final version to be published and agree to be accountable for all aspects of the work in ensuring that questions related to the accuracy or integrity of any part of the work are appropriately investigated and resolved. EKO was involved interpreting the data for the work and revising the work critically for important intellectual content, approved the final version to be published and agrees to be accountable for all aspects of the work in ensuring that questions related to the accuracy or integrity of any part of the work are appropriately investigated and resolved. LM, FU, MB and KWS made substantial contributions to the conception and design of the work. They were involved in drafting the work, approved the final version to be published and agree to be accountable for all aspects of the work in ensuring that questions related to the accuracy or integrity of any part of the work are appropriately investigated and resolved.

Funding: This study was supported by the National Institute for Health Research (NIHR) [Health Services and Delivery Research (project reference 16/52/25)]. The views expressed are those of the authors and not necessarily those of the NIHR or the Department of Health and Social Care.

For peer review only - http://bmjopen.bmj.com/site/about/guidelines.xhtml

Competing interests: None declared.

Ethics approval: This study was approved by the London Stanmore ethics committee, reference 17/LO/2102

Data sharing statement: Data are available upon reasonable request from the corresponding author subject to restrictions to preserve anonymity and personal privacy [JC]. These data are not publicly available as they contain information that could compromise research participant privacy/consent. Data will be available beginning 1 year and ending 5 years after publication to researchers who propose a methodologically sound proposal. Proposals should be directed to j.chudleigh@city.ac.uk. To gain access, data requesters will need to sign a data access agreement.

For peer review only

For peer review only - http://bmjopen.bmj.com/site/about/guidelines.xhtml

REFERENCES

1. UK Newborn Screening Programme Centre. Health Professional Handbook: A guide to newborn blood spot screening for healthcare professionals. London: UK Newborn Screening Programme Centre, 2012:1-56.
2. Bush A. Newborn screening for cystic fibrosis – benefit or bane? *Paediatric respiratory reviews* 2008;9:301-2.
3. Public Health England. Newborn Blood Spot Screening Programme in the UK: Data Collection and Performance Analysis Report 2016 to 2017. London, 2018:1-65.
4. Public Health England. NHS Sickle Cell and Thalassaemia Screening Programme Data Report 2015/16: Trends and performance analysis. London, 2017:1-81.
5. Public Health England. Newborn blood spot screening: programme handbook: Public Health England, 2018.
6. Salm A, Yetter E, Tluczek A. Informing parents about positive newborn screening results: Parents' recommendations *Journal of Child Health Care* 2012;16(4):367-81.
7. Ulph F, Cullinan T, Qureshi N, et al. Parents' responses to receiving sickle cell or cystic fibrosis carrier results for their child following newborn screening. *Eur J Hum Genet* 2015;23(4):459-65. doi: DOI 10.1038/ejhg.2014.126
8. Ulph F, Cullinan T, Qureshi N, et al. The impact on parents of receiving a carrier result for sickle cell or cystic fibrosis for their child via newborn screening. *Eur J Hum Genet* 2014;22
9. Tluczek A, Clark R, McKechnie AC, et al. Factors affecting parent-child relationships one year after positive newborn screening for cystic fibrosis or congenital hypothyroidism. *J Dev Behav Pediatr* 2015;36(1):24-34. doi: 10.1097/DBP.0000000000000112
10. Chudleigh J, Buckingham S, Dignan J, et al. Parents' Experiences of Receiving the Initial Positive Newborn Screening (NBS) Result for Cystic Fibrosis and Sickle Cell Disease. *J Genet Couns* 2016 doi: 10.1007/s10897-016-9959-4
11. Parker H, Qureshi N, Ulph F, et al. Imparting carrier status results detected by universal newborn screening for sickle cell and cystic fibrosis in England: a qualitative study of current practice and policy challenges. *BMC Health Serv Res* 2007;7:203. doi: 1472-6963-7-203 [pii] 10.1186/1472-6963-7-203 [published Online First: 2007/12/15]
12. Ulph F, Cullinan T, Qureshi N, et al. Parents' responses to receiving sickle cell or cystic fibrosis carrier results for their child following newborn screening. *Eur J Hum Genet* 2015;23(4):459-65. doi: 10.1038/ejhg.2014.126
13. Chudleigh J, Ren CL, Barben J, et al. International approaches for delivery of positive newborn bloodspot screening results for CF. *Journal of cystic fibrosis : official journal of the European Cystic Fibrosis Society* 2019;18(5):614-21. doi: 10.1016/j.jcf.2019.04.004
14. Kai J, Ulph F, Cullinan T, et al. Communication of carrier status information following universal newborn screening for sickle cell disorders and cystic fibrosis: qualitative study of experience and practice. *Health Technol Assess* 2009;13(57):1-82, iii. doi: 10.3310/hta13570 [published Online First: 2009/12/02]
15. Tluczek A, Koscik RL, Farrell PM, et al. Psychosocial risk associated with newborn screening for cystic fibrosis: parents' experience while awaiting the sweat-test appointment. *Pediatrics* 2005;115(6):1692-703. doi: 10.1542/peds.2004-0275
16. Collins JL, La Pean A, O'Tool F, et al. Factors that influence parents' experiences with results disclosure after newborn screening identifies genetic carrier status for cystic fibrosis or sickle cell hemoglobinopathy. *Patient Educ Couns* 2012 doi: S0738-3991(11)00606-9 [pii] 10.1016/j.pec.2011.12.007 [published Online First: 2012/01/14]
17. Buchbinder M, Timmermans S. Newborn screening for metabolic disorders: parental perceptions of the initial communication of results. *Clin Pediatr (Phila)* 2012;51(8):739-44. doi: 0009922812446011 [pii] 10.1177/0009922812446011 [published Online First: 2012/05/09]

For peer review only - http://bmjopen.bmj.com/site/about/guidelines.xhtml

18. DeLuca JM, Kearney MH, Norton SA, et al. Parents' experiences of expanded newborn screening evaluations. *Pediatrics* 2011;128(1):53-61. doi: peds.2010-3413 [pii] 10.1542/peds.2010-3413 [published Online First: 2011/06/29]
19. Rueegg CS, Barben J, Hafen GM, et al. Newborn screening for cystic fibrosis - The parent perspective. *Journal of cystic fibrosis : official journal of the European Cystic Fibrosis Society* 2016;15(4):443-51. doi: 10.1016/j.jcf.2015.12.003
20. Chudleigh JC, H. Bonham, J.R. Olander, E.K. Moody, L. Simpson, A. Morris, S. Ulph, F. Bryon, M. Southern, K.W. . A qualitative exploration of parents' experiences of receiving positive newborn bloodspot screening results for nine conditions in England. *In Press* 2020
21. Chudleigh J, Bonham J, Bryon M, et al. Rethinking Strategies for Positive Newborn Screening Result (NBS+) Delivery (ReSPoND): a process evaluation of co-designing interventions to minimise impact on parental emotional well-being and stress. *Pilot Feasibility Stud* 2019;5:108. doi: 10.1186/s40814-019-0487-5
22. Braun V, Clarke V. Using thematic analysis in psychology. *Qualitative Research in Psychology* 2006;3(2):77-101.
23. Milford C, Kriel Y, Njau I, et al. Teamwork in Qualitative Research: Descriptions of a Multicountry Team Approach. *International Journal of Qualitative Methods* 2017;16:1-10.
24. DeCuir-Gunby JT, Marshall PL, McCulloch AW. Developing and Using a Codebook for the Analysis of Interview Data: An Example from a Professional Development Research Project. *Field Methods* 2011;23(2):136-55.
25. Marsh VM, Kamuya DM, Molyneux SS. 'All her children are born that way': gendered experiences of stigma in families affected by sickle cell disorder in rural Kenya. *Ethn Health* 2011;16(4-5):343-59. doi: 10.1080/13557858.2010.541903
26. Moody L, Atkinson L, Kehal I, et al. Healthcare professionals' and parents' experiences of the confirmatory testing period: a qualitative study of the UK expanded newborn screening pilot. *BMC Pediatr* 2017;17(1):121. doi: 10.1186/s12887-017-0873-1
27. Gerada C. Doctors and suicide. *Br J Gen Pract* 2018;68(669):168-69. doi: 10.3399/bjgp18X695345
28. Selby A. More than 300 overworked NHS nurses have died by suicide in just seven years. *Daily Mirror* 2019.
29. Gilbey P. Qualitative analysis of parents' experience with receiving the news of the detection of their child's hearing loss. *Int J Pediatr Otorhinolaryngol* 2010;74(3):265-70. doi: 10.1016/j.ijporl.2009.11.017

For peer review only

For peer review only - http://bmjopen.bmj.com/site/about/guidelines.xhtml

Consolidated criteria for reporting qualitative studies (COREQ): 32-item checklist

Developed from:
Tong A, Sainsbury P, Craig J. Consolidated criteria for reporting qualitative research (COREQ): a 32-item checklist for interviews and focus groups. *International Journal for Quality in Health Care*. 2007. Volume 19, Number 6: pp. 349 – 357

YOU MUST PROVIDE A RESPONSE FOR ALL ITEMS. ENTER N/A IF NOT APPLICABLE

No. Item	Guide questions/description	Reported on Page #
**Domain 1: Research team and reflexivity**		
Personal Characteristics		
1. Inter viewer/facilitator	Which author/s conducted the inter view or focus group?	Section 2.5
2. Credentials	What were the researcher's credentials? E.g. PhD, MD	PhD
3. Occupation	What was their occupation at the time of the study?	Children's Nurse (JC) and Psychologist (HC)
4. Gender	Was the researcher male or female?	Female
5. Experience and training	What experience or training did the researcher have?	MSc Research Methods; extensive clinical experience; interview techniques.
Relationship with participants		
6. Relationship established	Was a relationship established prior to study commencement?	No
7. Participant knowledge of the interviewer	What did the participants know about the researcher? e.g. personal goals, reasons for doing the research	Professional role – Children's nurse, researcher role in this study
8. Interviewer characteristics	What characteristics were reported about the inter viewer/facilitator? e.g. Bias, assumptions, reasons and interests in the research topic	Section 5.0
**Domain 2: study design**		
Theoretical framework		
9. Methodological orientation and Theory	What methodological orientation was stated to underpin the study? e.g. grounded theory, discourse analysis, ethnography, phenomenology, content analysis	Qualitative exploratory design
Participant selection		

For peer review only - http://bmjopen.bmj.com/site/about/guidelines.xhtml

10. Sampling	How were participants selected? e.g. purposive, convenience, consecutive, snowball	Purposive
11. Method of approach	How were participants approached? e.g. face-to-face, telephone, mail, email	Email
12. Sample size	How many participants were in the study?	Seventeen
13. Non-participation	How many people refused to participate or dropped out? Reasons?	Three – time constraints / workload demands
Setting		
14. Setting of data collection	Where was the data collected? e.g. home, clinic, workplace	Workplace
15. Presence of non-participants	Was anyone else present besides the participants and researchers?	No
16. Description of sample	What are the important characteristics of the sample? e.g. demographic data, date	Staff involved in communicating the initial positive NBS result in the preceding 6 months.
Data collection		
17. Interview guide	Were questions, prompts, guides provided by the authors? Was it pilot tested?	Yes
18. Repeat interviews	Were repeat inter views carried out? If yes, how many?	N/A
19. Audio/visual recording	Did the research use audio or visual recording to collect the data?	Audio recording
20. Field notes	Were field notes made during and/or after the inter view or focus group?	Yes
21. Duration	What was the duration of the inter views or focus group?	37 minutes (range 19-58 minutes)
22. Data saturation	Was data saturation discussed?	Yes
23. Transcripts returned	Were transcripts returned to participants for comment and/or correction?	No but a staff meeting was held after all interviews had been conducted as per the protocol
**Domain 3: analysis and findings**		
Data analysis		
24. Number of data coders	How many data coders coded the data?	Two
25. Description of the coding tree	Did authors provide a description of the coding tree?	Yes
26. Derivation of themes	Were themes identified in advance or derived from the data?	Derived from the data
27. Software	What software, if applicable, was used to manage the data?	N/A
28. Participant checking	Did participants provide feedback on the findings?	Yes

For peer review only

Reporting		
29. Quotations presented	Were participant quotations presented to illustrate the themes/findings? Was each quotation identified? e.g. participant number	Results section
30. Data and findings consistent	Was there consistency between the data presented and the findings?	Yes
31. Clarity of major themes	Were major themes clearly presented in the findings?	Results
32. Clarity of minor themes	Is there a description of diverse cases or discussion of minor themes?	Discussion

Once you have completed this checklist, please save a copy and upload it as part of your submission. When requested to do so as part of the upload process, please select the file type: *Checklist*. You will NOT be able to proceed with submission unless the checklist has been uploaded. Please DO NOT include this checklist as part of the main manuscript document. It must be uploaded as a separate file.

For peer review only
